# Pathogenic LRRK2 regulates centrosome cohesion via Rab10/RILPL1-mediated CDK5RAP2 displacement

**DOI:** 10.1016/j.isci.2022.104476

**Published:** 2022-05-30

**Authors:** Elena Fdez, Jesús Madero-Pérez, Antonio J. Lara Ordóñez, Yahaira Naaldijk, Rachel Fasiczka, Ana Aiastui, Javier Ruiz-Martínez, Adolfo López de Munain, Sally A. Cowley, Richard Wade-Martins, Sabine Hilfiker

**Affiliations:** 1 Institute of Parasitology and Biomedicine "López-Neyra", Consejo Superior de Investigaciones Científicas (CSIC), 18016 Granada, Spain; 2 Department of Anesthesiology, Department of Pharmacology, Physiology and Neuroscience, New Jersey Medical School, Rutgers, The State University of New Jersey, Newark, NJ 07103, USA; 3 CIBERNED (Institute Carlos III), Madrid, Spain; 4 Cell Culture Platform, Biodonostia Institute, San Sebastian, Spain; 5 Department of Neurology, Hospital Universitario Donostia-OSAKIDETZA, San Sebastian, Spain; 6 Neurosciences Area, Biodonostia Institute, San Sebastian, Spain; 7 Department of Neurosciences, University of the Basque Country, San Sebastian, Spain; 8 Sir William Dunn School of Pathology, University of Oxford, Oxford, UK; 9 Oxford Parkinson's Disease Centre, Department of Physiology, Anatomy and Genetics, University of Oxford, Oxford, UK; 10 Department of Physiology, Anatomy and Genetics, University of Oxford, Oxford, UK

**Keywords:** Biological sciences, Neuroscience, Cellular neuroscience, Cell biology, Functional aspects of cell biology

## Abstract

Mutations in LRRK2 increase its kinase activity and cause Parkinson's disease. LRRK2 phosphorylates a subset of Rab proteins which allows for their binding to RILPL1. The phospho-Rab/RILPL1 interaction causes deficits in ciliogenesis and interferes with the cohesion of duplicated centrosomes. We show here that centrosomal deficits mediated by pathogenic LRRK2 can also be observed in patient-derived iPS cells, and we have used transiently transfected cell lines to identify the underlying mechanism. The LRRK2-mediated centrosomal cohesion deficits are dependent on both the GTP conformation and phosphorylation status of the Rab proteins. Pathogenic LRRK2 does not displace proteinaceous linker proteins which hold duplicated centrosomes together, but causes the centrosomal displacement of CDK5RAP2, a protein critical for centrosome cohesion. The LRRK2-mediated centrosomal displacement of CDK5RAP2 requires RILPL1 and phospho-Rab proteins, which stably associate with centrosomes. These data provide fundamental information as to how pathogenic LRRK2 alters the normal physiology of a cell.

## Highlights

• The Parkinson's disease kinase LRRK2 causes a centrosomal cohesion deficit
• The cohesion deficit depends on the Rab GTP conformation and phosphorylation status
• The cohesion deficit is not because of the displacement of centrosomal linker proteins
• The deficit is because of CDK5RAP2 displacement mediated by the RILPL1/phospho-Rab8/10 complex

## Graphical abstract

**Figure:**
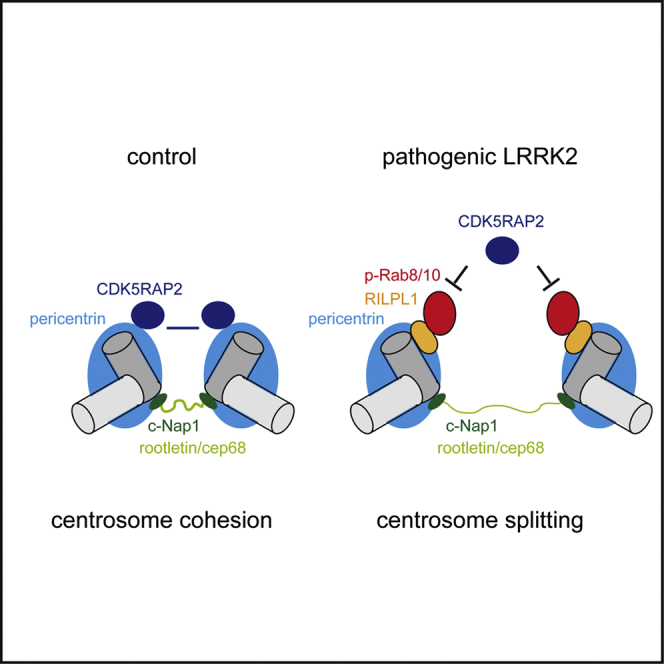

## Introduction

Point mutations in Leucine rich repeat kinase 2 (LRRK2) cause autosomal-dominant familial Parkinson's disease (PD). Pathogenic LRRK2 phosphorylates a subset of Rab proteins (Steger et al., 2016), small GTPases which function as key regulators for intracellular membrane trafficking events (Hutagalung and Novick, 2011; Pfeffer, 2013). Rab8 and Rab10 act as prominent LRRK2 kinase substrates, and upon their phosphorylation both gain the ability to bind to RILPL1 (Steger et al., 2017). Pathogenic LRRK2 blocks primary cilia formation in cultured cells and in certain areas of the mouse brain, which is associated with the RILPL1-mediated centrosomal accumulation of phospho-Rab10 (Dhekne et al., 2018; Steger et al., 2017). This accumulation interferes with the recruitment of tau tubulin kinase 2 (TTBK2), thereby preventing the release of centriolar CP110 which is necessary for the initiation of cilia formation (Sobu et al., 2021).

We have previously shown that the LRRK2-mediated phosphorylation of Rab8 and Rab10 and their subsequent RILPL1-mediated centrosomal accumulation not only interferes with ciliogenesis, appropriate centrosome positioning and directional cell migration, but also with the cohesion of duplicated centrosomes in dividing cells (Lara Ordonez et al., 2019; Madero-Pérez et al., 2018). In peripheral cells from LRRK2-PD patients as compared to healthy controls, quantitative mass spectrometry-based assays can detect increased phospho-Rab10 levels (Karayel et al., 2020), and centrosomal cohesion deficits serve as a sensitive cellular biomarker for enhanced LRRK2 kinase activity (Fernandez et al., 2019). However, neither of those assays can be easily translated into clinical settings, requiring the need for further mechanistic insights into these LRRK2 kinase-mediated cellular alterations.

Duplicated centrosomes gradually mature during the G2 phase of the cell cycle and remain connected by a proteinaceous linker until the onset of mitosis to assure appropriate centrosome cohesion (Agircan et al., 2014; Mardin and Schiebel, 2012). The filamentous linker proteins rootletin and cep68 hold the duplicated centrosomes together by binding to the centrosomal docking protein c-Nap1 (Bahe et al., 2005; Graser et al., 2007; Vlijm et al., 2018; Yang et al., 2006). At the onset of mitosis, the mitotic kinase Polo-like kinase 1 (Plk1) activates Nek2a, which in turn phosphorylates linker and docking components to cause linker disassembly, and these Nek2a-mediated effects are counteracted by protein phosphatase 1α (PP1α) (Faragher and Fry, 2003; Fry et al., 1998a, 1998b; Mardin et al., 2010, 2011; Meraldi and Nigg, 2001; Mi et al., 2007). In addition, CDK5RAP2 (also called CEP215) and pericentrin are critical for appropriate centrosome cohesion (Graser et al., 2007), even though they do not function as linker proteins and are not displaced via Nek2a-mediated phosphorylation.

In the present work, we have investigated the mechanism by which pathogenic LRRK2 causes centrosomal cohesion deficits in cultured cells, and we report that the mechanism requires Rab10 and RILPL1 proteins and is due to interfering with the proper centrosomal localization of CDK5RAP2.

## Results

### Pathogenic LRRK2 causes centrosomal cohesion deficits in human iPS cells

**Figure 1:**
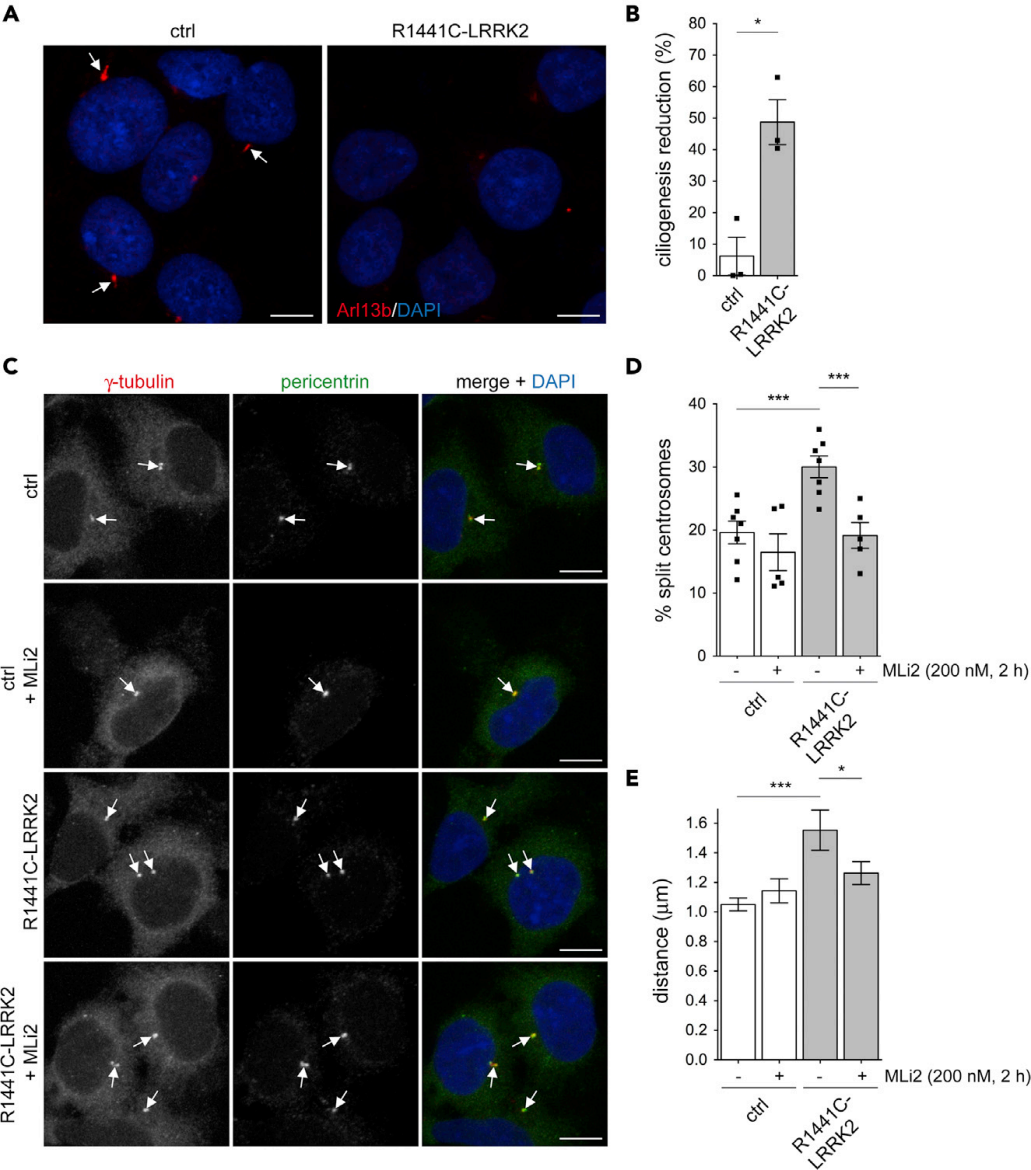
Pathogenic LRRK2 causes ciliary and centrosomal cohesion deficits in patient-derived iPSCs (A) Example of undifferentiated healthy patient-derived LRRK2^WT/WT^ (control) or gene-edited LRRK2^R1441C/WT^ (R1441C) iPSCs stained for Arl13b (red) and DAPI (blue). Arrows point to elongated, Arl13b-positive cilia. Scale bar, 10 μm. (B) Quantification of the percentage reduction in ciliated cells in control and gene-edited R1441C iPSCs. Around 200 cells were quantified per experiment. Bars represent mean ± SEM (n = 3 independent experiments; p = 0.01); ∗p < 0.05. (C) Example of control or R1441C iPSCs treated in the absence or presence of MLi2 (200 nM, 2 h), and stained for γ-tubulin (green), pericentrin (red) and DAPI (blue). Arrows point to centrosomes as identified by co-staining with the two centrosomal markers. Scale bar, 10 μm. (D) Quantification of the percentage of control or R1441C iPSCs with split centrosomes (duplicated centrosomes with a distance between their centers >1.5 μm), in the presence or absence of 200 nM MLi2 for 2 h before immunocytochemistry as indicated. Around 150–200 cells were quantified per condition per experiment. Bars represent mean ± SEM (n = five to seven independent experiments; ctrl versus R1441C-LRRK2, p = 0.001; R1441C-LRRK2 versus R1441C-LRRK2 + MLi2: p = 0.002); ∗∗∗p < 0.005. (E) Same as in (D), but depicting the mean distance between duplicated centrosomes. Around 50 cells with duplicated centrosomes per condition were analyzed. Bars represent mean ± SEM (ctrl versus R1441C-LRRK2, p = 0.002; R1441C-LRRK2 versus R1441C-LRRK2 + MLi2: p = 0.024); ∗∗∗p < 0.005; ∗p < 0.05.

Previous studies have reported LRRK2-mediated ciliary deficits in undifferentiated induced pluripotent stem cells (iPSCs) carrying the LRRK2 G2019S mutation as compared to the corresponding zinc finger-corrected wild-type cell line (Dhekne et al., 2018). To determine whether a ciliary phenotype could also be observed in iPSCs carrying other pathogenic LRRK2 mutations, we generated an isogenic iPSC line carrying the R1441C mutation using CRISPR/Cas9 (LRRK2^R1441C/WT^) (Figure S1) on top of a healthy control iPSC line (Fernandes et al., 2016). When undifferentiated cells were plated onto geltrex-coated coverslips and stained for the presence of cilia, cells carrying the R1441C mutation showed around 40–50% less ciliation than the corresponding wild-type cells, similar to what has been previously described for LRRK2 G2019S cells (Dhekne et al., 2018) (Figures 1A and 1B). The LRRK2 R1441C mutant iPSCs also showed a centrosomal cohesion deficit as compared to the corresponding wild-type cells, as evidenced either by the percentage of cells with duplicated centrosomes larger than 1.5 microns apart (Figures 1C and 1D) or by the average distance between duplicated centrosomes (Figure 1E). The centrosomal deficit in the LRRK2 R1441C iPSCs was fully restored upon transient treatment of cells with the LRRK2 kinase inhibitor MLi2 (Figures 1C–1E). In addition, a centrosomal cohesion deficit was also observed in neuronal precursor cells (NPCs) differentiated from LRRK2 G2019S iPSCs as compared to NPCs differentiated from the gene-corrected isogenic control iPS line (Figure S2). These experiments show that distinct pathogenic LRRK2 mutations cause centrosomal cohesion deficits also in human iPSCs and in iPSC-derived NPCs.

### The LRRK2-mediated centrosomal cohesion deficits depend on both the GTP conformation and phosphorylation status of Rab8a

**Figure 2:**
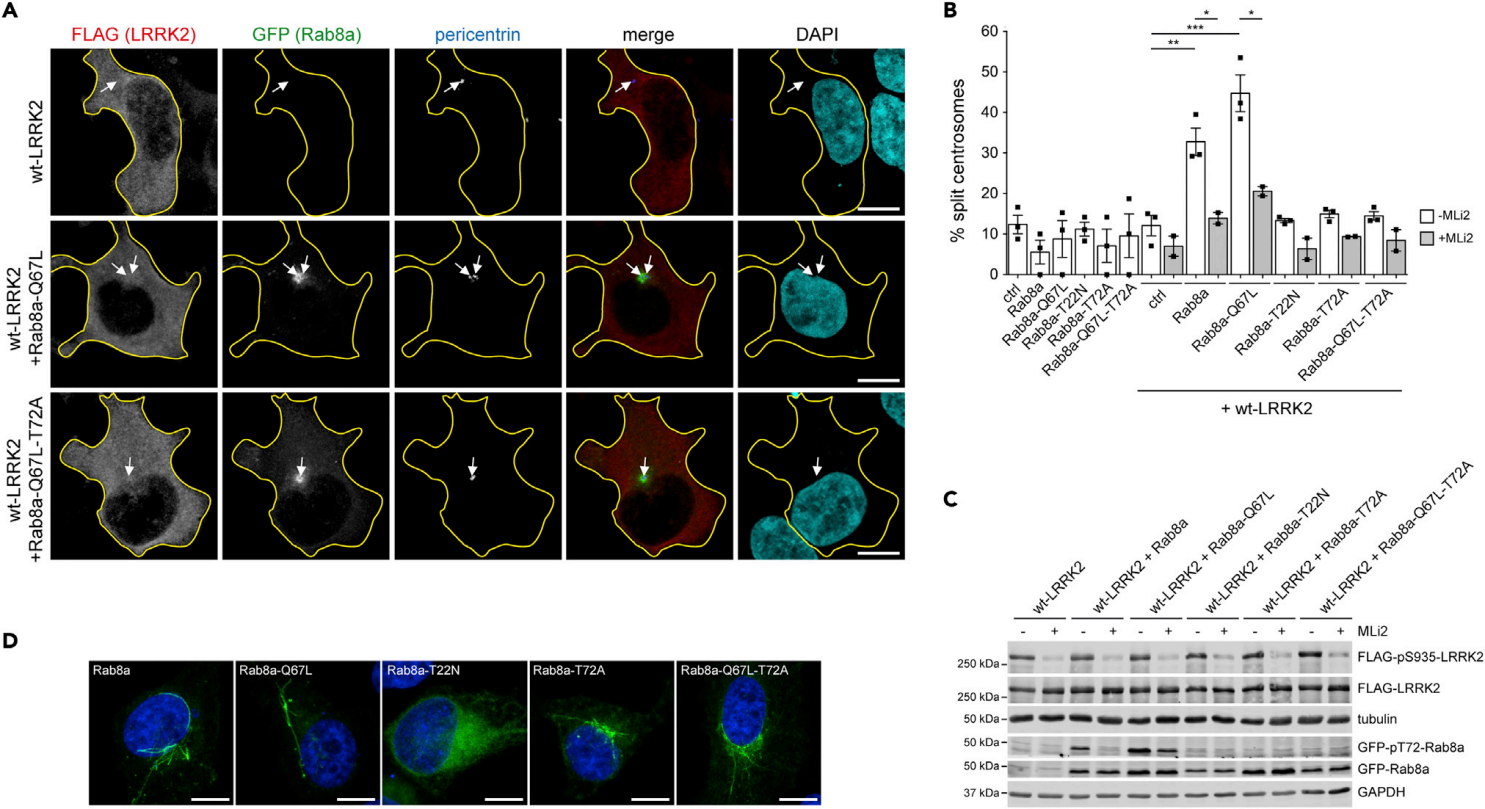
LRRK2-mediated centrosomal cohesion deficits depend on both GTP conformation and phosphorylation status of Rab8a (A) Example of HEK293T cells co-transfected with FLAG-tagged wild-type LRRK2 and GFP-tagged Rab8a-Q67L (GTP-trapped) or GFP-tagged Rab8a-Q67L-T72A (GTP-trapped but non-phosphorylatable), and stained with antibody against FLAG (red) and the centrosomal marker pericentrin (pseudocolored blue) and DAPI. Arrows point to centrosomes in transfected cells. Cell boundaries (yellow) are shown and were determined by FLAG staining because of FLAG-tagged wild-type LRRK2 expression (or GFP because of GFP-tagged Rab8a expression for single transfection experiments). Scale bar, 10 μm. (B) Quantification of the split centrosome phenotype in cells expressing the indicated constructs, in either the absence or presence of LRRK2 kinase inhibitor MLi2 (100 nM, 2 h) as indicated. Around 100–150 cells were quantified per condition and experiment. Bars represent mean ± SEM (n = two to three independent experiments; wt-LRRK2 versus wt-LRRK2 + Rab8a, p = 0.007; wt-LRRK2 + Rab8a versus wt-LRRK2 + Rab8a + MLi2, p = 0.024; wt-LRRK2 versus wt-LRRK2 + Rab8a-Q67L, p = 0.003; wt-LRRK2 + Rab8a-Q67L versus wt-LRRK2 + Rab8a-Q67L + MLi2, p = 0.026); ∗∗∗p < 0.005; ∗∗p < 0.01; ∗p < 0.05. (C) Cells were co-transfected with FLAG-tagged wild-type LRRK2 and GFP-tagged Rab8a constructs as indicated, left untreated or incubated with 100 nM MLi2 for 2 h, and extracts blotted for FLAG-tagged LRRK2 (FLAG-LRRK2), FLAG-tagged S935-phosphorylated LRRK2 (FLAG-pS935-LRRK2) as a readout for on-target effect of MLi2, phosphorylated GFP-tagged Rab8a (GFP-pT72-Rab8a), total GFP-tagged Rab8a (GFP-Rab8a), and tubulin or GAPDH as loading controls. (D) HeLa cells were transfected with the indicated GFP-tagged Rab8a constructs, briefly fixed, washed and stained with DAPI. All GFP-tagged Rab8a constructs except the inactive T22N mutant display a localization consistent with their presence in a tubular early recycling compartment. Scale bar, 10 μm.

The LRRK2-mediated phosphorylation of Rab8a (on T72) and Rab10 (on T73) allows for their interaction with a new set of effector proteins including RILPL1 (Steger et al., 2017), which causes their accumulation at/around the centrosome and correlates with the centrosomal cohesion and ciliogenesis deficits (Dhekne et al., 2018; Lara Ordonez et al., 2019; Madero-Pérez et al., 2018). Recent studies indicate that such interactions may also depend on the GTP-bound conformation of the phosphorylated Rab proteins (Waschbüsch et al., 2020). We therefore analyzed whether the LRRK2-mediated centrosomal cohesion deficits were dependent on both the GTP conformation and phosphorylation status of Rab8a. We coexpressed FLAG-tagged wild-type LRRK2 with either GFP-tagged wild-type Rab8a, Rab8a-Q67L (GTP-trapped conformation) or Rab8a-T22N (GDP-bound conformation) in HEK293T cells, respectively. As previously described (Madero-Pérez et al., 2018), co-expression of FLAG-tagged wild-type LRRK2 with GFP-tagged wild-type Rab8a to increase the amount of phosphorylated-tagged Rab8a caused a centrosomal cohesion phenotype (Figures 2A and 2B). Co-expression of FLAG-tagged wild-type LRRK2 with GFP-tagged Rab8a-Q67L also caused a pronounced cohesion deficit, whereas no effects were observed when coexpressing Rab8a-T22N or a GTP-trapped Rab8a mutant unable to be phosphorylated by LRRK2 (Rab8a-Q67L-T72A) (Figures 2A and 2B). The centrosomal deficits mediated by co-expression of FLAG-tagged LRRK2 with GFP-tagged wild-type or GTP-trapped Rab8a correlated with an increase in the phosphorylation status of the transiently expressed tagged Rab8a proteins (Figure 2C) and were at least in part reverted by the LRRK2 kinase inhibitor MLi2 (Figure 2B). In addition, all GFP-tagged mutants except the GDP-bound version of Rab8a were expressed to similar degrees and were properly membrane-associated (Figures 2C and 2D). These data indicate that the LRRK2-mediated centrosomal cohesion deficits depend on both the phosphorylation and nucleotide-bound status of Rab8a.

We wondered whether co-expression of GTPase activating proteins (GAPs) to decrease the GTP-bound status of the endogenous phospho-Rab proteins may reduce the cohesion deficits mediated by pathogenic LRRK2. TBC1D4/AS160 displays GAP activity toward Rab10 *in vivo* (Sano et al., 2007), and both TBC1D17 and TBC1D30 have been reported to display GAP activity toward Rab8a (Fuchs et al., 2007; Yoshimura et al., 2007). Co-expression of GFP-tagged TBC1D30 did not alter the centrosomal cohesion deficits mediated by FLAG-tagged pathogenic LRRK2 (Figures S3A and S3B). Similarly, expression of active or inactive GFP-tagged TBC1D4 or TBC1D17 constructs only marginally decreased the pathogenic LRRK2-mediated centrosomal cohesion deficits (Figures S3A and S3C). None of the three TBC proteins were able to decrease the LRRK2-mediated phosphorylation levels of endogenous Rab8a or Rab10 as assessed by Western blotting (Figure S3D) or the localization of phospho-Rab10 as assessed by immunocytochemistry (Figure S3E). Thus, GAP protein expression is unable to modulate the phosphorylation status or centrosomal cohesion deficits mediated by pathogenic LRRK2.

### Phospho-Rab8a and phospho-Rab10 accumulate on centrosomes in the presence of pathogenic LRRK2

To determine whether the phospho-Rabs stably interact with centrosomes, we biochemically purified fractions enriched for centrosomes from HEK293T cells. Centrosomes were isolated by centrifugation using a discontinuous sucrose gradient, and fractions were immunoblotted for centrosomal markers, with the centrosome fraction eluting at around 55% sucrose as previously described (Gogendeau et al., 2015) (Figure S4A). To enhance detection of the Rab proteins, we transiently expressed FLAG-tagged Rab8a or FLAG-tagged Rab10. Although neither total nor phosphorylated Rab8a were detectable on centrosome-enriched fractions in the absence of LRRK2 expression, co-expression of FLAG-Rab8a with pathogenic LRRK2 resulted in the presence of phospho-Rab8a on centrosomes (Figure S4B). Similarly, phospho-Rab10 was detected on centrosomes in the presence, but not in the absence of pathogenic LRRK2 expression (Figure S4C). Although the identity of the Rab8a/10-containing vesicular compartment in contact with the centrosome remains unknown, these data are consistent with the notion that both membrane-bound Rab8a and Rab10 stably associate with centrosomes upon their LRRK2-mediated phosphorylation.

### Regulation of LRRK2-mediated centrosomal cohesion deficits by the Plk1/Nek2/linker cascade

**Figure 3:**
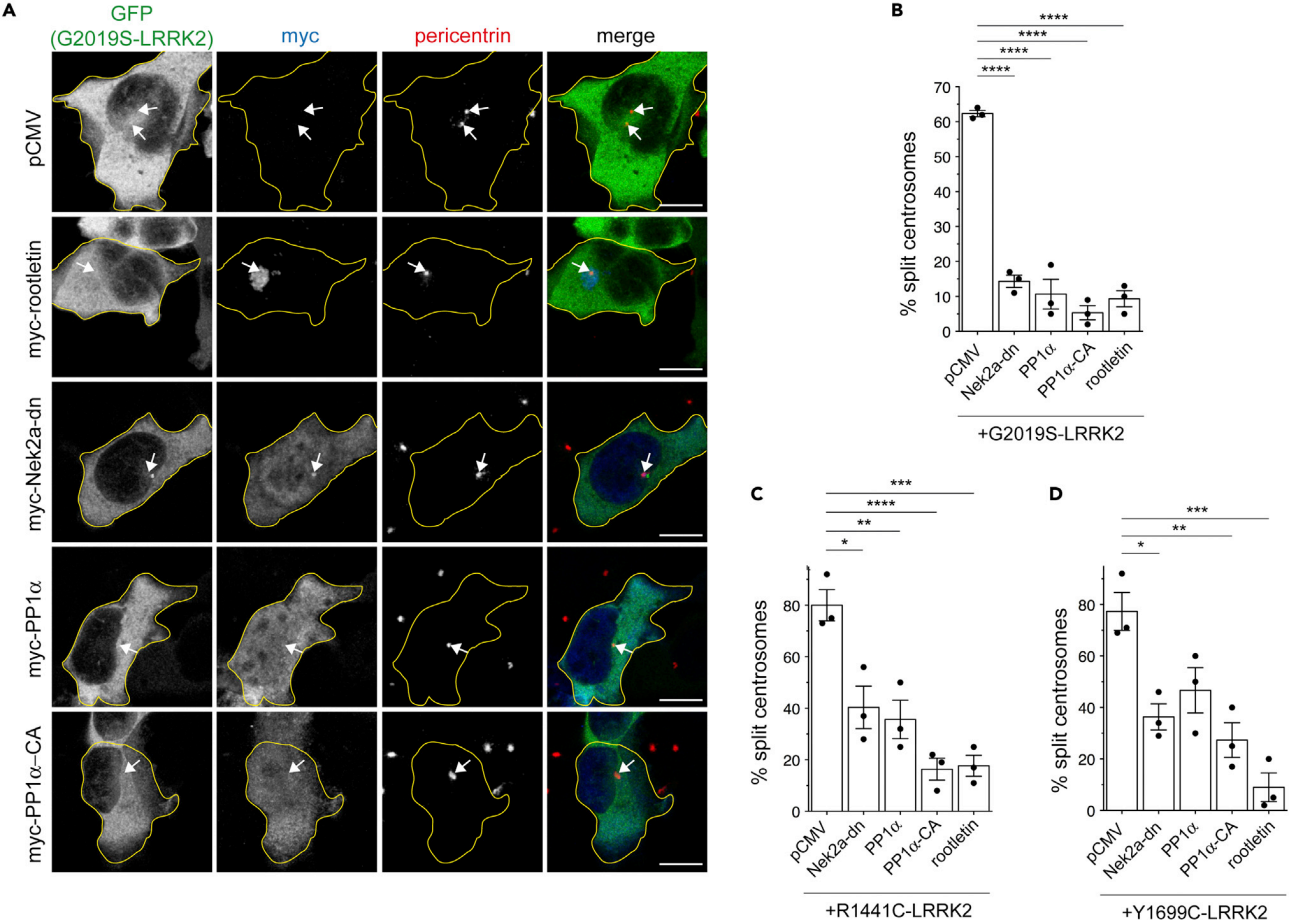
Rescue of LRRK2-mediated cohesion deficits by expression of centrosomal linker protein rootletin or by Nek2a/PP1α modulation (A) Example of HEK293T cells co-transfected with GFP-tagged G2019S-LRRK2 and with pCMV, or myc-tagged rootletin, inactive dominant-negative Nek2a-K37R (Nek2a-dn), PP1α or catalytically active PP1α-T320A (PP1-CA), and stained with anti-myc and anti-pericentrin antibodies. Arrows point to centrosomes in transfected cells. Cell boundaries (yellow) are shown and were determined because of GFP-tagged G2019S-LRRK2 expression. Scale bar, 10 μm. (B) Quantification of the split centrosome phenotype in cells transfected with GFP-tagged G2019S-LRRK2 along with the indicated myc-tagged constructs. Around 30 transfected cells with duplicated centrosomes were analyzed for each condition. Bars represent mean ± SEM (n = 3 independent experiments; p < 0.001 in all cases); ∗∗∗∗p < 0.001. (C) Same as in (B), but cells transfected with GFP-tagged R1441C-LRRK2 along with indicated myc-tagged constructs. Bars represent mean ± SEM (n = 3 independent experiments; R1441C-LRRK2 versus R1441C-LRRK2 + Nek2a-dn, p = 0.017; R1441C-LRRK2 versus R1441C-LRRK2 + PP1α, p = 0.009; R1441C-LRRK2 versus R1441C-LRRK2 + PP1α-CA, p < 0.001; R1441C-LRRK2 versus R1441C-LRRK2 + rootletin, p = 0.001); ∗∗∗∗p < 0.001; ∗∗∗p < 0.005; ∗∗p < 0.01; ∗p < 0.05. (D) Same as in (B), but cells transfected with GFP-tagged Y1699C-LRRK2 along with indicated myc-tagged constructs. Bars represent mean ± SEM (n = 3 independent experiments; Y1699C-LRRK2 versus Y1699C-LRRK2 + Nek2a-dn, p = 0.010; Y1699C-LRRK2 versus Y1699C-LRRK2 + PP1α-CA, p = 0.007; Y1699C-LRRK2 versus Y1699C-LRRK2 + rootletin, p = 0.001); ∗∗∗p < 0.005; ∗∗p < 0.01; ∗p < 0.05.

We next wondered whether the centrosomal cohesion deficits are subject to regulation by linker proteins known to hold duplicated centrosomes together in a manner dependent on their phosphorylation status (Agircan et al., 2014; Mardin and Schiebel, 2012). Transient overexpression of Nek2a or treatment of cells with the phosphatase inhibitor calyculin A caused an increase in the percentage of cells with split duplicated centrosomes (Figures S5A and S5B). This was associated with an increase in the percentage of transfected cells in G2/M phase and a concomitant decrease of cells in G1 phase (Figure S5C), even though not leading to cell cycle arrest, as previously described (Faragher and Fry, 2003; Fry et al., 1998b; Mayor et al., 2000). Similarly, transient overexpression of distinct pathogenic LRRK2 mutants caused an increase in the percentage of transfected cells in G2/M and a decrease in G1 phase (Figures S5D–S5F), and specifically an increase in the percentage of cells in G2 as assessed by cyclinB staining (Figure S5G). Such cell cycle alterations were observed with pathogenic LRRK2, but not with a kinase-inactive point mutant, suggesting that they are mediated by the LRRK2 kinase activity (Figure S5H). The cohesion deficit mediated by the expression of various pathogenic LRRK2 mutants was fully reverted when coexpressing the linker protein rootletin (Figures 3A–3D). Furthermore, co-expression of wild type or constitutively active PP1α (Xu et al., 2003), or co-expression of inactive, dominant-negative Nek2a (Meraldi and Nigg, 2001) reverted the centrosomal cohesion deficit mediated by distinct pathogenic LRRK2 mutants (Figures 3A–3D), whereas these constructs were without effect when expressed on their own (Figures S6A–S6C). In A549 cells, where overexpression levels are more modest as compared to HEK293T cells (Figure S7A), FLAG-tagged pathogenic LRRK2 expression also caused centrosomal cohesion deficits, which were partially reverted when co-expressing these constructs (Figures S7B–S7D). In addition, acute treatment of cells with a Plk1 inhibitor partially reverted the centrosomal cohesion deficits induced by either Nek2a or by pathogenic LRRK2 expression (Figures S8A–S8C), whereas inhibitors of p38, a member of the mitogen-activated protein kinase (MAPK) family and a regulator of the G2/M transition (Lee et al., 2010) were without effect (Figures S8D and S8E). Thus, the centrosomal cohesion deficits in pathogenic LRRK2-expressing cells are not merely a reflection of cell cycle alterations but are subject to regulation by the well-known cascade involving Plk1, Nek2a, and the proteinaceous linker between duplicated centrosomes.

### Pathogenic LRRK2 causes centrosomal displacement of CDK5RAP2

**Figure 4:**
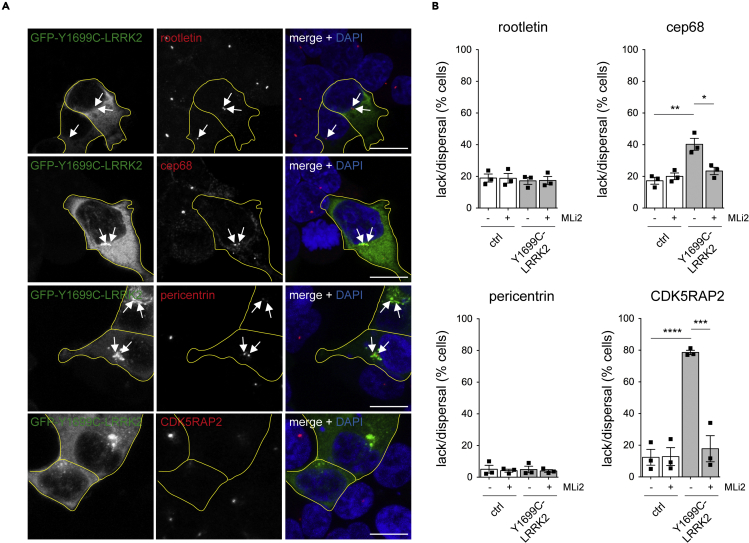
Pathogenic LRRK2 expression causes loss/dispersal of CDK5RAP2 staining (A) Example of HEK293T cells transfected with GFP-tagged Y1699C-LRRK2 and stained for proteinaceous centrosomal linker proteins rootletin and cep68, for pericentrin or for CDK5RAP2 along with DAPI. Arrows point to staining in transfected cells. Cell boundaries (yellow) are shown and were determined because of GFP-tagged Y1699C-LRRK2 expression. Please note that certain transiently expressed GFP-tagged pathogenic LRRK2 mutants can display a cytosolic and additional dot-like or filamentous-like localization, as previously described (Blanca Ramírez et al., 2017; Godena et al., 2014; Kett et al., 2012; Watanabe et al., 2020). Scale bar, 10 μm. (B) Quantification of the percentage of control or GFP-Y1699C-LRRK2 transfected cells displaying a lack of staining with antibodies against various proteins as indicated, either in the presence or absence of 100 nM MLi2 for 2 h before processing. Around 100–150 transfected cells were scored per condition and experiment. Bars represent mean ± SEM (n = 3 independent experiments; cep68: ctrl versus Y1699C-LRRK2, p = 0.006; Y1699C-LRRK2 versus Y1699C-LRRK2 + MLi2, p = 0.019; CDK5RAP2: ctrl versus Y1699C-LRRK2, p < 0.001; Y1699C-LRRK2 versus Y1699C-LRRK2 + MLi2, p = 0.001); ∗∗∗∗p < 0.001; ∗∗∗p < 0.005; ∗∗p < 0.01; ∗p < 0.05.

Because Nek2a-mediated phosphorylation of centrosomal linker proteins causes their displacement followed by centrosome separation (Bahe et al., 2005; Yang et al., 2006), we reasoned that pathogenic LRRK2 may function by a similar mechanism. Surprisingly, although Nek2a overexpression was associated with pronounced loss of rootletin staining in transfected cells (Figures S9A and S9B), this was not observed in cells expressing pathogenic LRRK2 (Figures 4A and 4B). Similarly, Nek2a expression caused pronounced loss of cep68 (Figures S9A and S9B), another centrosomal linker protein which is part of the rootletin network (Graser et al., 2007), but only a slight displacement was observed upon pathogenic LRRK2 expression (Figures 4A and 4B). Co-staining with a centrosomal marker confirmed that these proteins were normally localized to centrosomes in non-transfected as well as in LRRK2-transfected cells (Figure S10A).

Apart from structural linker proteins, an RNAi screen also identified CDK5RAP2 as being required for proper centrosome cohesion (Graser et al., 2007). CDK5RAP2 is not implicated in forming the linker structure, but displays centrosomal localization throughout mitosis and is thought to influence linker integrity indirectly by a currently unknown mechanism (Agircan et al., 2014; Graser et al., 2007). Strikingly, although expression of Nek2a did not alter CDK5RAP2 staining (Figures S9A and S9B), pathogenic LRRK2 caused the dispersal/disappearance of CDK5RAP2 staining in around 80% of transfected cells, which was reversed upon acute treatment of cells with the LRRK2 inhibitor MLi2, indicating that it was LRRK2 kinase activity-mediated (Figures 4A and 4B). Co-staining with a centrosomal marker confirmed that CDK5RAP2 was displaced from centrosomes also as judged by measuring integrated fluorescence density (Figure S10B). In contrast, staining for the pericentriolar matrix protein pericentrin was present in virtually all interphase cells, whether transfected with Nek2 or with pathogenic LRRK2, respectively (Figures S9, 4 and S10). The LRRK2-mediated displacement/dispersal of CDK5RAP2 was also detected when staining with another anti-CDK5RAP2 antibody (Figure S11). In addition, displacement of CDK5RAP2 was observed in nearly 70% of A549 cells transfected with FLAG-tagged pathogenic LRRK2 and was reverted upon MLi2 treatment, indicating that it was neither cell-type nor tag-dependent (Figures S12A and S12B). As judged by immunoblot analysis (Figures S7A and S12C), CDK5RAP2 levels were not affected by pathogenic LRRK2 expression, suggesting that the relative inability to detect CDK5RAP2 by immunofluorescence is because of its dissociation from centrosomes rather than its degradation.

**Figure 5:**
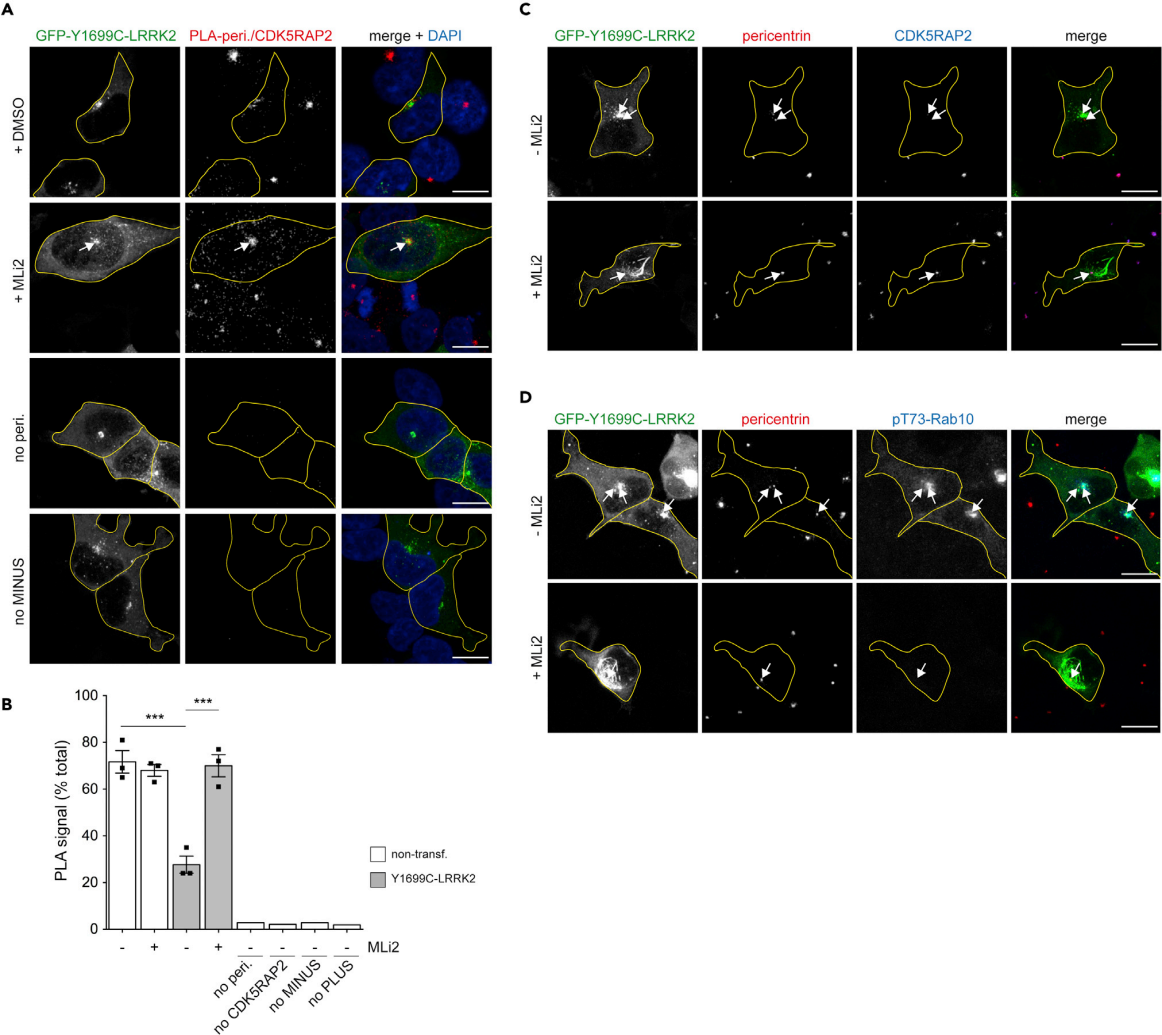
Pathogenic LRRK2 decreases proximity ligation signals between pericentrin and CDK5RAP2 (A) Example of proximity ligation signal in HEK293T cells transfected with GFP-tagged Y1699C-LRRK2, treated with or without MLi2 (100 nM, 2 h) before processing for PLA and stained with DAPI. Arrow points to a strong dot-like PLA signal in the transfected cell upon MLi2 treatment. Cell boundaries (yellow) are shown and were determined because of GFP-tagged Y1699C-LRRK2 expression. Two control reactions (omission of anti-pericentrin antibody or MINUS probe, respectively) did not display visible PLA signal. Scale bar, 10 μm. (B) Quantification of the percentage of non-transfected or GFP-Y1699C-LRRK2 transfected cells displaying an intense dot-like PLA signal. The four PLA control reactions included omission of primary antibodies or of PLA probes, respectively. Bars represent mean ± SEM (n = 3 independent experiments; non-transfected versus Y1699C-LRRK2, p = 0.001; Y1699C-LRRK2 versus Y1699C-LRRK2 + MLi2, p = 0.002); ∗∗∗p < 0.005. (C) Example of HEK293T cells transfected with GFP-tagged Y1699C-LRRK2 and treated with or without MLi2 (100 nM, 2 h) before processing for immunocytochemistry with rabbit anti-CDK5RAP2 and mouse anti-pericentrin antibodies. Arrows point to centrosomes in transfected cells. Scale bar, 10 μm. (D) Same as in (C), but cells stained with mouse anti-pericentrin and rabbit anti-pT73-Rab10 antibodies. Arrows point to centrosomes in transfected cells. Scale bar, 10 μm.

**Figure 6:**
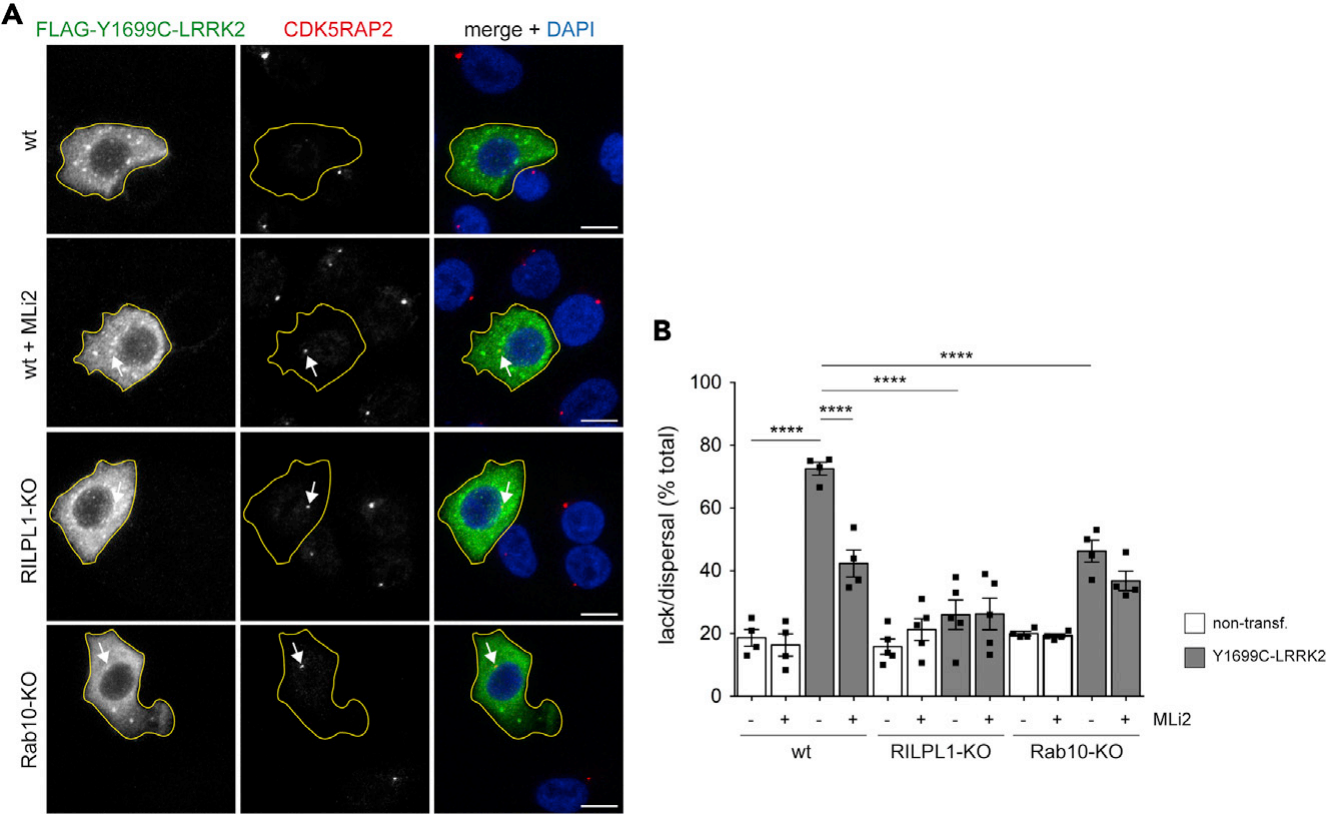
Loss of Rab10 or RILPL1 restores centrosomal CDK5RAP2 levels in pathogenic LRRK2-expressing cells (A) Example of wild-type (wt) A549 cells, or RILPL1 knockout (KO) or Rab10-KO cells transfected with FLAG-tagged Y1699C-mutant LRRK2 and treated with or without MLi2 (200 nM, 2h) where indicated before staining for CDK5RAP2 and DAPI. Arrows point to CDK5RAP2 staining in transfected cells. Cell boundaries (yellow) are shown and were determined by FLAG staining because of FLAG-tagged Y1699C-LRRK2 expression. Scale bar, 10 μm. (B) Quantification of the percentage of non-transfected or Y1699C LRRK2-transfected wild-type, RILPL1-KO or Rab10-KO cells displaying a lack/dispersal of staining with anti-CDK5RAP2 antibody. Around 40–50 transfected cells were scored per condition and experiment. Bars represent mean ± SEM (n = four to five independent experiments; p < 0.001 in all cases); ∗∗∗∗p < 0.001.

**Figure 7:**
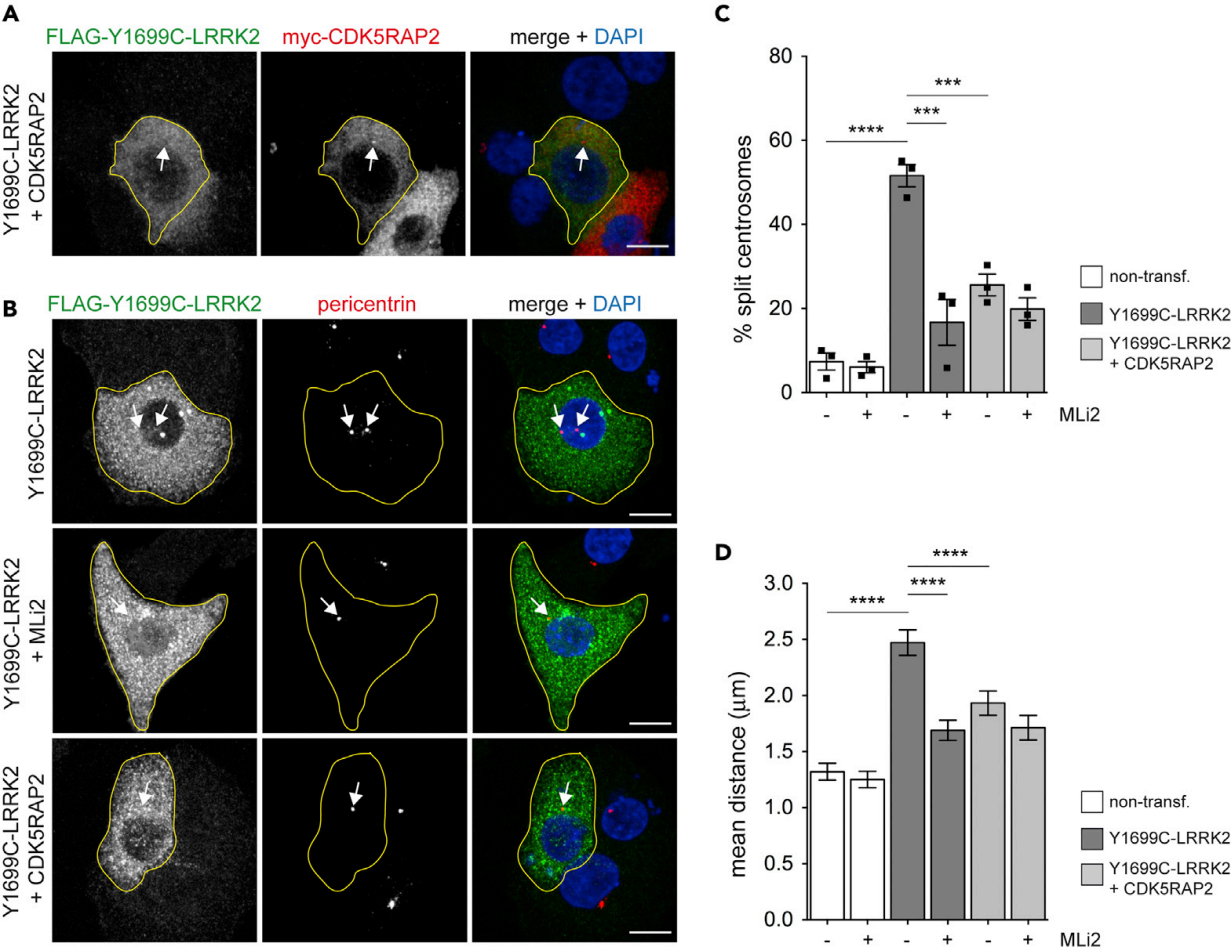
Rescue of LRRK2-mediated cohesion deficits by expression of CDK5RAP2 (A) Example of wild-type A549 cells co-transfected with FLAG-tagged Y1699C-LRRK2 and myc-tagged CDK5RAP2 and stained with antibodies against LRRK2, myc, and DAPI. Arrow points to perinuclear accumulation of myc-tagged CDK5RAP2. Scale bar, 10 μm. (B) Example of A549 cells co-transfected with FLAG-tagged Y1699C-LRRK2 and myc-tagged CDK5RAP2, and stained with antibodies against FLAG, pericentrin, and DAPI. Arrows point to centrosomes in transfected cells. Scale bar, 10 μm. (C) Quantification of the split centrosome phenotype in non-transfected cells, or in cells either co-transfected with FLAG-Y1699C-LRRK2 and empty pCMV vector or with FLAG-Y1699C-LRRK2 and myc-CDK5RAP2 vector as indicated, in either the absence or presence of LRRK2 kinase inhibitor (200 nM, 2 h). Around 50 transfected cells were quantified per condition and experiment. Bars represent mean ± SEM (n = 3 independent experiments; non-transfected versus Y1699C-LRRK2, p < 0.001; Y1699C-LRRK2 versus Y1699C-LRRK2 + MLi2, p = 0.004; Y1699C-LRRK2 versus Y1699C-LRRK2 + CDK5RAP2, p = 0.002); ∗∗∗∗p < 0.001; ∗∗∗p < 0.005. (D) Same as in (C), but depicting the mean distances between duplicated centrosomes. The distances between duplicated centrosomes were measured from around 150 cells per condition. Bars represent mean ± SEM (p < 0.001 in all cases); ∗∗∗∗p < 0.001.

As another means to determine whether pathogenic LRRK2 expression interferes with the proper centrosomal localization of CDK5RAP2, we analyzed the spatial proximity between endogenous CDK5RAP2 and pericentrin by performing *in situ* proximity ligation assays (PLA), which allow for the sensitive detection of endogenous protein interactions (Söderberg et al., 2006). Pathogenic LRRK2 expression led to a significant decrease in the percentage of cells displaying a proximity ligation signal between CDK5RAP2 and pericentrin (Figures 5A and 5B), which correlated with the centrosomal displacement of CDK5RAP2 (Figure 5C) and with the prominent centrosomal accumulation of phosphorylated Rab10, in a manner reverted upon MLi2 treatment (Figure 5D). We next tested whether the LRRK2-mediated CDK5RAP2 displacement requires endogenous Rab10 and RILPL1 proteins. Pathogenic LRRK2 expression caused prominent loss of endogenous CDK5RAP2 staining in A549 wild-type cells but not in cells deficient in either RILPL1 or Rab10 (Figures 6A and 6B), indicating that it causes the centrosomal cohesion deficits by CDK5RAP2 displacement via Rab phosphorylation and RILPL1 binding. Finally, we evaluated whether expression of CDK5RAP2 may modulate the pathogenic LRRK2-mediated centrosomal cohesion deficits. Over 95% of transfected A549 wild-type cells displayed co-expression of pathogenic LRRK2 and myc-tagged CDK5RAP2, which was localized to the cytosol but also accumulating at the centrosome in the majority of co-transfected cells (Figure 7A). Co-expression of CDK5RAP2 with pathogenic LRRK2 reverted the LRRK2-mediated centrosomal cohesion deficits both as assessed by measuring the percentage of split duplicated centrosomes or the mean distance between duplicated centrosomes (Figures 7C and 7D). Altogether, these results indicate that pathogenic LRRK2 triggers Rab10 phosphorylation and RILPL1 binding which displaces CDK5RAP2, a protein critical for the proper cohesion of duplicated centrosomes.

**Figure 8:**
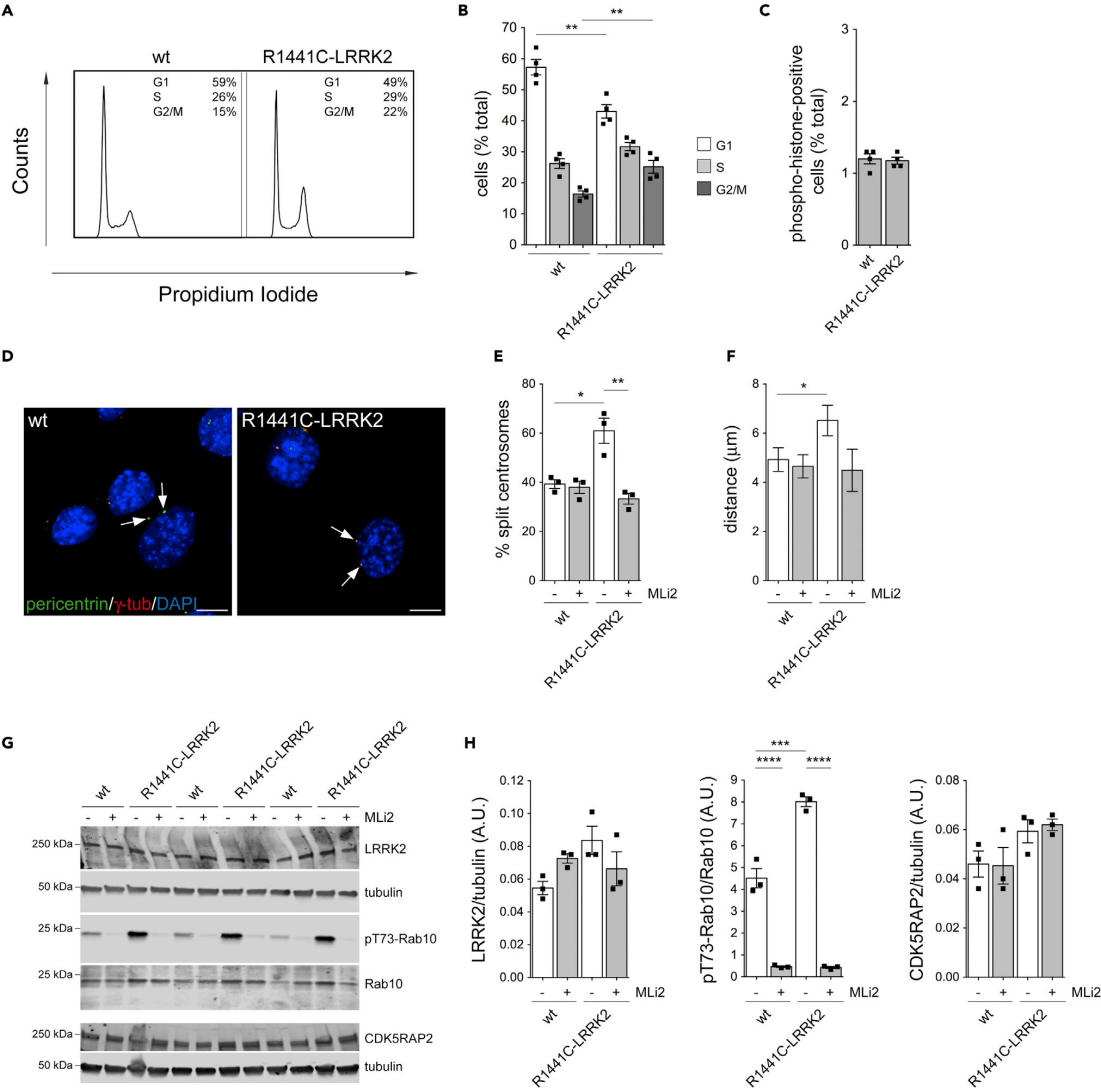
Cell cycle alterations and centrosomal cohesion deficits in R1441C-LRRK2 knockin MEF cells (A) Representative FACS traces from wild-type and R1441C-LRRK2 knockin MEFs, with the percentage of cells in each phase of the cell cycle indicated to the right of each histogram. (B) Quantification of experiments of the type depicted in (A). Bars represent mean ± SEM (n = 4 independent experiments; G1: wt versus R1441C-LRRK2, p = 0.005; G2/M: wt versus R1441C-LRRK2, p = 0.008); ∗∗p < 0.01. (C) Cells were stained with a fluorescently labeled anti-phospho-histone H3 antibody and analyzed using FACS to determine the percentage of cells in M phase. Bars represent mean ± SEM (n = 4 independent experiments). (D) Representative images of wild-type or R1441C-LRRK2 MEFs stained with antibodies against two centrosomal markers (pericentrin and γ-tubulin) and with DAPI. Arrows point to duplicated centrosomes. Scale bar, 10 μm. (E) Quantification of the split centrosome phenotype in wild-type and R1441C-LRRK2 MEFs either treated with DMSO or with MLi2 (200 nM, 2 h) before processing for immunocytochemistry. Bars represent mean ± SEM (n = 3 independent experiments; wt versus R1441C-LRRK2, p = 0.016; R1441C-LRRK2 versus R1441C-LRRK2 + MLi2, p = 0.007); ∗∗p < 0.01; ∗p < 0.05. (F) Quantification of the distances between duplicated centrosomes in the indicated cells in the absence or presence of MLi2. Bars represent mean ± SEM (n = 30–90 cells with duplicated centrosomes; wt versus R1441C-LRRK2, p = 0.041); ∗p < 0.05. (G) Wild type or R1441C-LRRK2 MEFs were treated with DMSO or MLi2 (200 nM, 2 h), and extracts blotted for LRRK2, phospho-Rab10 (pT73-Rab10), Rab10, CDK5RAP2, or tubulin as loading controls. (H) Quantification of LRRK2/tubulin, pT73-Rab10/Rab10 and CDK5RAP2/tubulin levels from Western blots depicted in (G). Bars depict mean ± SEM (n = 3 independent samples per genotype; wt versus wt + MLi2, p < 0.001; wt versus R1441C-LRRK2, p = 0.002; R1441C-LRRK2 versus R1441C-LRRK2 + MLi2, p < 0.001); ∗∗∗∗p < 0.001; ∗∗∗p < 0.005.

To probe for the observed phenotypes in cells expressing endogenous levels of LRRK2, we employed mouse embryonic fibroblasts (MEFs) derived from homozygous R1441C-LRRK2 knockin mice and littermate matched wild-type controls (Steger et al., 2017). As compared to wild-type MEFs, R1441C-LRRK2 MEFs displayed an increase in G2 and a concomitant decrease in G1 phase of the cell cycle (Figures 8A–8C). The R1441C-LRRK2 MEFs displayed a centrosomal cohesion deficit as assessed by an increase in the mean distance between duplicated centrosomes, which was reverted upon MLi2 treatment (Figures 8D–8F). These alterations correlated with a kinase activity-mediated increase in the levels of phospho-Rab10, without changes in the total levels of CDK5RAP2 as judged by immunoblotting (Figures 8G and 8H).

**Figure 9:**
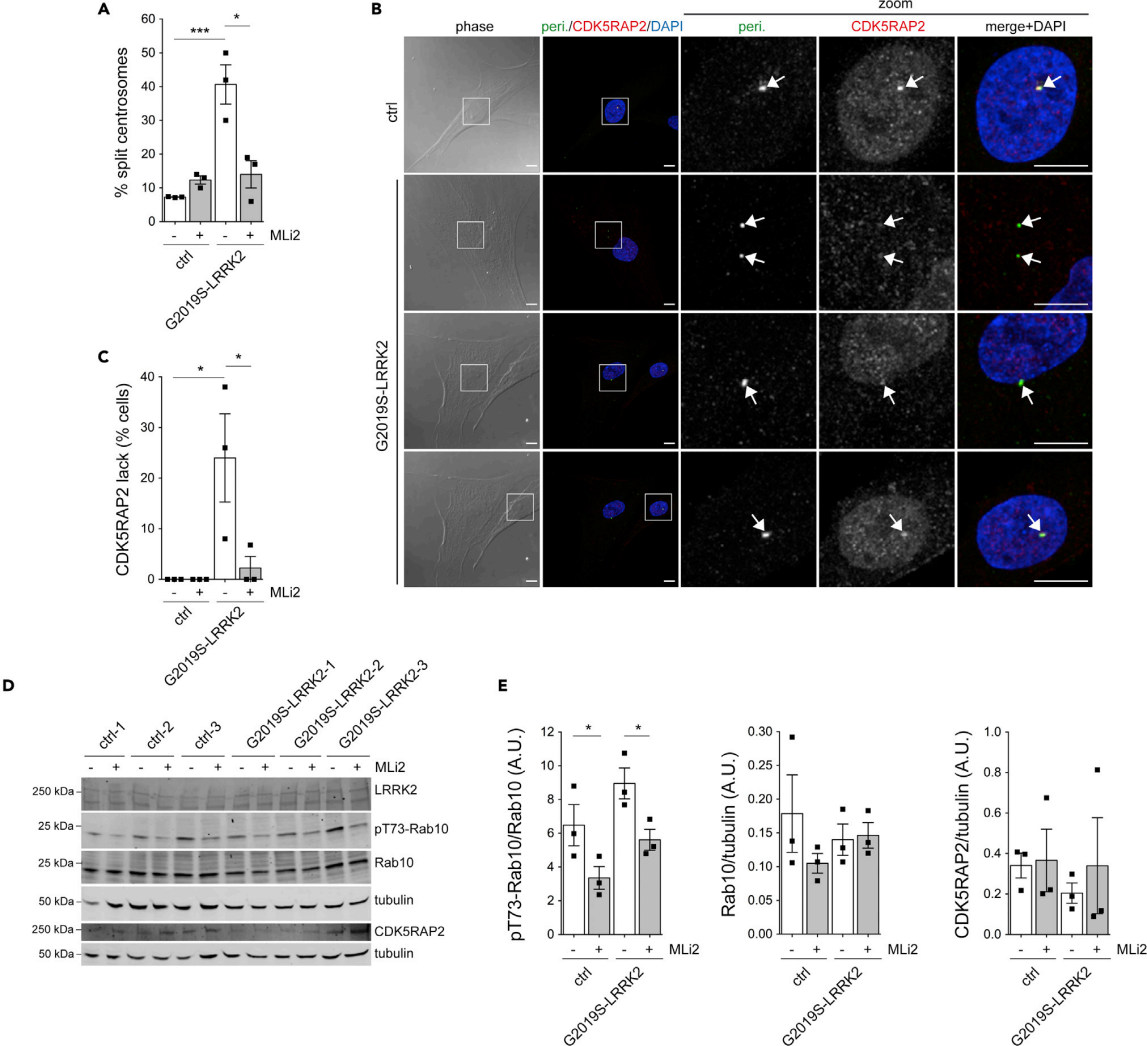
Centrosomal cohesion deficits and CDK5RAP2 displacement in primary human dermal fibroblasts from G2019S mutant LRRK2 PD patients as compared to healthy controls (A) Quantification of centrosomal cohesion deficits in primary fibroblasts treated with DMSO or with MLi2 (200 nM, 2 h) from 100 cells for each of three control and three G2019S LRRK2 PD patients. Bars represent mean ± SEM (between three independent lines; ctrl versus G2019S-LRRK2, p = 0.004; G2019S-LRRK2 versus G2019S-LRRK2 + MLi2, p = 0.019). ∗∗∗p < 0.005; ∗p < 0.05. (B) Example of control (ctrl) and G2019S LRRK2 PD patient primary fibroblasts stained with antibodies against pericentrin, CDK5RAP2, and DAPI. Arrows point to centrosomes as determined by pericentrin staining. Note decreased intensity of centrosomal CDK5RAP2 staining in G2019S-LRRK2 fibroblasts in cells with duplicated centrosomes as well as in cells with one centrosome. Scale bar, 10 μm. Scale bar in zoomed images, 10 μm. (C) Quantification of integrated density of centrosomal CDK5RAP2 staining in a circle of 1.6 μm diameter placed on top of centrosome as identified by pericentrin staining in fibroblasts from three control and three G2019S LRRK2 PD patients, with background intensity adjacent to the centrosome subtracted in all cases. Quantification was performed on 50–70 individual cells per condition and genotype, and the percentage of cells not displaying any CDK5RAP2 staining determined for each line. Bars represent mean ± SEM (between three independent lines each; ctrl versus G2019S-LRRK2, p = 0.032; G2019S-LRRK2 versus G2019S-LRRK2 + MLi2, p = 0.049). ∗p < 0.05. (D) Primary fibroblasts from three control and three G2019S LRRK2 PD patients were treated with DMSO or MLi2 (200 nM, 2 h), and extracts blotted for LRRK2, phospho-Rab10 (pT73-Rab10), Rab10, CDK5RAP2, or tubulin as loading controls. (E) Quantification of pT73-Rab10/Rab10, Rab10/tubulin and CDK5RAP2/tubulin levels from Western blots depicted in (D). Bars depict mean ± SEM (n = 3 independent lines per genotype; ctrl versus G2019S-LRRK2, p = 0.036; G2019S-LRRK2 versus G2019S-LRRK2 + MLi2, p = 0.031); ∗p < 0.05.

The antibody suitable for detection of murine CDK5RAP2 in MEF cells was not compatible with immunocytochemistry approaches to probe for alterations in the centrosomal localization of CDK5RAP2. Therefore, we next employed primary human dermal fibroblasts from healthy control and G2019S LRRK2-PD patients (Madero-Pérez et al., 2018). Centrosomal cohesion deficits were observed in primary fibroblasts from G2019S LRRK2-PD patients as compared to healthy controls, and were reverted by short-term application of MLi2 (Figure 9A). A significant percentage of G2019S LRRK2-PD fibroblasts displayed a kinase-mediated loss of centrosomal CDK5RAP2 as compared to healthy controls (Figures 9B and 9C), which correlated with a modest nonsignificant increase in the levels of phospho-Rab10, without changes in the total levels of CDK5RAP2 as judged by immunoblotting (Figures 9D and 9E). Thus, endogenous levels of pathogenic LRRK2 also cause centrosomal cohesion deficits and displacement of centrosomal CDK5RAP2.

## Discussion

We show here that the centrosomal cohesion deficits mediated by pathogenic LRRK2 depend on the GTP conformation and phosphorylation status of membrane-bound Rab8a. A Rab8a mutant mimicking the GDP-bound state is cytosolic, unable to be phosphorylated and unable to trigger the LRRK2-mediated centrosomal cohesion deficits. Conversely, a non-phosphorylatable Rab8a mutant mimicking the GTP-bound conformation is membrane-bound yet unable to trigger the LRRK2-mediated cohesion deficits. These data indicate that the Rab protein needs to be membrane-bound and GTP-bound to be phosphorylated by LRRK2 and cause the centrosomal cohesion deficits. However, overexpression of GAP proteins for either Rab8 or Rab10 did not reverse the LRRK2-mediated Rab phosphorylation or centrosomal cohesion deficits. These data are consistent with structural studies predicting a steric clash mediated by the phospho-residue on Rab8a which impairs its interaction with GAPs (Waschbüsch et al., 2020), and with experimental data showing that substitutions which mimic the LRRK2 phosphorylation site in Rab10 interfere with its interaction with the GAP TBC1D4/AS160 (Liu et al., 2018). Thus, strategies aimed at decreasing the GTP-bound status of membrane-bound Rab8/10 are unable to modulate the downstream effects of LRRK2-phosphorylated Rab8/10.

Rootletin forms a proteinaceous linker which holds the duplicated centrosomes together and which is displaced upon Plk1-mediated Nek2a activation and phosphorylation. We show here that overexpression of this linker protein reverts the LRRK2-mediated centrosomal cohesion deficits. In addition, overexpression of a dominant-negative version of Nek2a or overexpression of PP1α to revert the Nek2a-mediated linker phosphorylation reverts the centrosomal cohesion deficits mediated by pathogenic LRRK2. However, and in contrast to Nek2a expression, pathogenic LRRK2 does not cause the displacement of centrosomal linker proteins. These data indicate that although the Nek2a-linker cascade can modulate the centrosomal cohesion deficits mediated by pathogenic LRRK2, it is not its primary target. Indeed, there exist at least two mechanisms assuring centrosome cohesion, which may explain why enhancing cohesion by overexpressing components related to the proteinaceous linker pathway can overcome deficits observed because of the centrosomal displacement of CDK5RAP2.

We have previously shown that LRRK2-phosphorylated Rab8 and Rab10 and its centrosomal binding partner RILPL1 are required for the process by which LRRK2 causes deficits in centrosomal cohesion (Lara Ordonez et al., 2019; Madero-Pérez et al., 2018). We show here that the LRRK2-phosphorylated Rab proteins associate with centrosomes, which correlates with the centrosomal displacement of CDK5RAP2 in a manner dependent on the presence of RILPL1. These data support a model in which the phospho-Rab/RILPL1 complex inhibits the proper centrosomal localization of CDK5RAP2, possibly by directly occluding the interaction of CDK5RAP2 with one of its reported centrosomal binding partners such as pericentrin (Pagan et al., 2015). In support of this notion, proximity ligation assays show a pronounced decrease of the CDK5RAP2-pericentrin signal which is reverted by LRRK2 kinase inhibitor.

The precise mechanism by which CDK5RAP2 regulates centrosomal cohesion is still unclear. Its persistence at the centrosome throughout mitosis makes it unlikely that it is part of a cell cycle-regulated linker structure. Rather, it may act in conjunction with pericentrin to regulate pericentriolar matrix and centrosome maturation (Graser et al., 2007; Kim and Rhee, 2014; Lee and Rhee, 2010), or assure additional control of centrosome cohesion via cytoskeletal components (Panic et al., 2015).

Loss of CDK5RAP2 causes reduced proliferation and premature differentiation of neural progenitor cells *in vitro* and in the intact mouse brain (Buchman et al., 2010; Kraemer et al., 2015; Lizarraga et al., 2010), and it will be interesting to determine whether the reported decrease in adult neurogenesis in LRRK2 G2019S transgenic mice, and the deficits in clonal expansion and differentiation of LRRK2 G2019S iPSC-derived neural progenitor cells are related to altered centrosomal CDK5RAP2 localization and concomitant cohesion deficits (Liu et al., 2012; Winner et al., 2011). Centrosomal cohesion deficits do not lead to cell-cycle arrest (Faragher and Fry, 2003; Fry et al., 1998a; Mayor et al., 2000), but can cause pronounced deficits in directional cell migration (Flanagan et al., 2017; Floriot et al., 2015), just as we and others have reported for pathogenic LRRK2-expressing cells (Caesar et al., 2013; Choi et al., 2015; Madero-Pérez et al., 2018). Importantly, future experiments aimed at determining alterations in the localization of CDK5RAP2 as an easy readout for enhanced LRRK2 kinase activity in patient-derived peripheral cells are warranted, as this may allow for the design of high-throughput patient stratification assays in clinical settings when employing LRRK2-related therapeutics.

### Limitations of the study

In this study, we have demonstrated the mechanism underlying the pathogenic LRRK2-mediated centrosomal cohesion deficits in various cell types. A limitation of this study is the absence of *in vivo* data to address the potential pathophysiological relevance of these alterations. However, cohesion deficits are observed in peripheral cells from LRRK2-PD patients, and centrosomal CDK5RAP2 displacement may serve as a proxy for increased LRRK2 kinase activity and amenable to high-throughput patient stratification purposes in clinical settings.

## STAR★Methods

### Key resources table

**Table:** 

REAGENT or RESOURCE	SOURCE	IDENTIFIER
**Antibodies**		

rabbit polyclonal anti-pericentrin	Abcam	Cat#ab4448; RRID: AB_304461
mouse monoclonal anti-pericentrin	Abcam	Cat#ab28144; RRID:AB_2160664
mouse monoclonal anti-γ-tubulin	Abcam	Cat#ab11316; RRID:AB_297920
mouse monoclonal anti-flag, clone M2	Sigma	Cat#F1804; RRID:AB_262044
mouse monoclonal anti-Myc, clone 9E10	Sigma	Cat#M4439; RRID:AB_439694
rabbit monoclonal anti-LRRK2	Abcam	Cat#ab133518
rabbit polyclonal anti-Arl13b	Proteintech	Cat#17711-1-AP; RRID:AB_2060867
mouse monoclonal anti-nestin	R and D Systems	Cat#mab1259 RRID:AB_2251304
rabbit polyclonal anti-Pax6	Covance	Cat#PRB-278P RRID:AB_291612
mouse monoclonal anti-Ki67	Biosciences	Cat#550609; RRID:AB_393778
mouse monoclonal anti-rootletin	Santa Cruz Biotechnology	Cat#sc-374056 RRID:AB_10918081
rabbit polyclonal anti-cep68	Proteintech	Cat#15147-1-AP; RRID:AB_2077081
rabbit polyclonal anti-cdk5rap2	Thermo Fisher	Cat#A300-554A; RRID:AB_477974
mouse monoclonal anti-cdk5rap2	Atlas Antibodies	Cat#AMAB91163; RRID:AB_2665827
rabbit polyclonal anti-cdk5rap2	Millipore	Cat#06-1398; RRID:AB_11203651
rabbit polyclonal anti-cNAP1	Proteintech	Cat#14498-1-AP; RRID:AB_2076918
rabbit polyclonal anti-ninein	Abcam	Cat#ab4447; RRID:AB_304460
rabbit polyclonal anti-cenexin	Abcam	Cat#ab43840; RRID:AB_880577
mouse monoclonal anti-cyclinB1	Santa Cruz Biotechnology	Cat#sc-245; RRID:AB_627338
rabbit polyclonal anti-rootletin	Prof. Erich Nigg	Bahe et al., 2005
rabbit polyclonal anti-GFP	Abcam	Cat#ab6556; RRID:AB_305564
mouse monoclonal anti-α-tubulin, clona DM1A	Sigma	Cat#T6199; RRID:AB_477583
mouse monoclonal anti-GAPDH	Santa Cruz Biotechnology	Cat#sc-32233; RRID:AB_627679
rabbit monoclonal anti-phospho-S935-LRRK2	Abcam	Cat#ab133450; RRID:AB_2732035
rabbit monoclonal anti-phospho-T72-Rab8a	Abcam	Cat#ab230260; RRID:AB_2814988
mouse monoclonal anti-Rab8	BD Biosciences	Cat#610844; RRID:AB_398163
rabbit monoclonal anti-phospho-T73-Rab10	Abcam	Cat#ab230261; RRID:AB_2811274
mouse monoclonal anti-Rab10	Sigma	Cat#SAB5300028
rabbit monoclonal anti-phospho-T73-Rab10	Abcam	Cat#ab241060; RRID:AB_2884876
rabbit polyclonal anti-cep290	Abcam	Cat#ab85728; RRID:AB_1859783
rabbit polyclonal anti-IFT88	Proteintech	Cat#13967-1-AP; RRID:AB_2121979
rabbit polyclonal anti-phosphohistone H3	Cell Signaling Technology	Cat#9716; RRID:AB_330212
mouse monoclonal anti-Rab11	BD Biosciences	Cat#610656; RRID:AB_397983
mouse monoclonal anti-LAMP1	Santa Cruz Biotechnology	Cat#sc-20011; RRID:AB_626853

**Bacterial and virus strains**		

MaxEfficiency DH5a competent cells	Thermo Fisher	Cat#11563117
XL-1blue supercompetent cells	Agilent Technologies	Cat#200236

**Chemicals, peptides, and recombinant proteins**		

MLi2	Abcam	Cat#ab254528
BI-2536	Medchem Express	Cat#HY-50698
SB203580	Millipore	Cat#559389
SB202474	Cayman Chemical	Cat#18749
SB202190	Axon Medchm	Cat#1364

**Critical commercial assays**		

Gibson Assemby Master Mix	New England Biolabs	Cat#E2611S
PureYield Plasmid Midiprep System	Promega	Cat#A2492
QuickChange II Site-Directed Mutagenesis Kit	Agilent Technologies	Cat#200523
Duolink *In Situ* PLA probe anti-rabbit PLUS	Sigma	Cat#DUO92002
Duolink *In Situ* PLA probe anti-mouse MINUS	Sigma	Cat#DUO92004
Duolink *In Situ* Detection Reagents Red	Sigma	Cat#DUO92008

**Experimental models: Cell lines**		

HEK293T	ATCC	Cat#CRL-3216
HeLa	ATCC	Cat#CRM-CCL-2
A549 wildtype cells	Prof. Dario Alessi	Ito et al., 2016
A549 Rab10-knockout cells	Prof. Dario Alessi	Dhekne et al., 2018
A549 RILPL1-knockout cells	Prof. Dario Alessi	Steger et al., 2017
iPS/SFC832-03-06	Prof. Richard Wade-Martins	Lee et al., 2020
iPS/SFC832-03-06 LRRK2 WT/WT C47	Prof. Richard Wade-Martins	Lee et al., 2020
iPS/SFC840-03-03	Prof. Richard Wade-Martins	Fernandes et al., 2016
iPS/SFC840-03-03 LRRK2wt/R1441C-H3	This study	N/A
MEF wildtype cells	Prof. Dario Alessi	Steger et al., 2017
MEF R1441C/R1441C knockin cells	Prof. Dario Alessi	Steger et al., 2017
healthy control primary human dermal fibroblasts	Prof. A. López de Munain	Gómez-Suaga et al., 2014
G2019S LRRK2-PD primary human dermal fibroblasts	Prof. A. López de Munain	Gómez-Suaga et al., 2014

**Recombinant DNA**		

pDEST53 GFP-hLRRK2 WT	Greggio et al., 2006	Addgene Cat#25044
pDEST53 GFP-hLRRK2 G2019S	Greggio et al., 2006	Addgene Cat#25045
pDEST53 GFP-hLRRK2 R1441C	Greggio et al., 2006	Addgene Cat#25046
pDEST53 GFP-hLRRK2 Y1699C	Greggio et al., 2006	Addgene Cat#25048
pDEST53 GFP-hLRRK2 K1906M	Blanca Ramírez et al., 2017	N/A
pCHMWS 3xflag-hLRRK2	Greggio et al., 2009	Prof. Elisa Greggio
pCHMWS 3xflag-hLRRK2 Y1699C	Greggio et al., 2009	Prof. Elisa Greggio
pGFP-hRab8a	Nachury et al., 2007	Addgene Cat#24898
pGFP-hRab8A Q67L	Nachury et al., 2007	Addgene Cat#24900
pGFP-hRab8a T22N	Nachury et al., 2007	Addgene Cat#24899
pGFP-hRab8a T72A	Madero-Pérez et al., 2018	N/A
pGFP-hRab8A Q67L-T72A	This study	N/A
GFP-hTBC1D30	Yoshimura et al., 2007	Prof. Francis Barr
GFP-hTBC1D4	Yoshimura et al., 2007	Prof. Francis Barr
GFP-hTBC1D4 R973K	Yoshimura et al., 2007	Prof. Francis Barr
GFP-hTBC1D17	Yoshimura et al., 2007	Prof. Francis Barr
GFP-hTBC1D17 R381A	Yoshimura et al., 2007	Prof. Francis Barr
pRcCMV-myc-rootletin	Bahe et al., 2005	Prof. Erich Nigg
pRcCMV-myc-Nek2	Fry et al., 1998a	Prof. Erich Nigg
pRcCMV-myc-Nek2 K37R	Fry et al., 1998a	Prof. Erich Nigg
pRcCMV-myc-cdk5rap2	Graser et al., 2007	Prof. Erich Nigg
pCMV-myc-PP1α	Xu et al., 2003	Prof. Len Neckers
pCMV-myc-PP1α T320A	Xu et al., 2003	Prof. Len Neckers

### Resource availability

#### Lead contact

Further information and requests for resources and reagents should be directed to and will be fulfilled by the lead contact, Sabine Hilfiker (sabine.hilfiker@rutgers.edu).

#### Materials availability

Plasmids and cell lines generated in this study are available upon request.

### Experimental model and subject details

#### Human iPSC lines and gene editing

Full information about the iPSC lines used in this study can be found in Table S1. The SFC840-03-03 LRKK2wt/R1441C-H3 line was generated by introducing the R1441C mutation on the SFC840-03-03 control line using CRISPR/Cas9 double nickase strategy followed by homology-directed repair with donor sequence. Two gRNAs targeting the desired sequences (Figure S1A) were cloned into a Cas9 nickase (Cas9-D10A nickase mutant) plasmid with puromycin selection (PX462; Addgene, #62987). Donor DNA template from the SFC840-03-03 control line was cloned into the pGEM®-T Easy vector (Promega) followed by the introduction of the R1441C mutation, a silent mutation in the PAM region to maximize the efficiency of gene-editing, and two silent mutations to create a unique *KpnI* restriction site for screening clones (Figure S1A). SFC840-03-03 iPS cells were nucleofected with the gRNA and donor DNA plasmids and selected with 1 μg/mL puromycin for 4 days. Surviving iPSCs were re-plated as single cells (15'000 cells in a 10 cm dish) and clones were screened by DNA sequencing and *KpnI* digestion. Positive clones were expanded, re-sequenced (Figure S1B), karyotyped and screened for a panel of common SNPs (Figure S1C), tested for pluripotency markers (Figure S1D) and common iPSC colony morphology (Figure S1E).

#### iPSC culture and NPC differentiation

Control and LRRK2 hiPSC lines were cultured in plates previously pre-coated with 1% geltrex (Thermofisher, A1413302) in KnockOut DMEM (Thermofisher, 10829-018) in cell culture media consisting of Essential 8^TM^ Medium (E8) (Thermofisher, A1517001), and were passaged as colonies with 0.5 mM EDTA (Thermofisher, 15575020) in PBS (Lonza, BE17-516F). For immunocytochemistry, cells were washed with PBS and treated with accutase (Thermofisher, A11105-01) for 5–10 min, and were collected with PBS and 10 μM Y-27632 ROCK inhibitor (Abcam, ab120129). After centrifugation for 5 min at 200 x g, cells were resuspended in E8 in the presence of 10 μM ROCK inhibitor, and were plated at a confluence of 50′000 cells/ml onto 1% geltrex pre-coated coverslips. Ciliogenesis was determined from cells grown in serum-free defined E8 medium with 10 μM ROCK inhibitor. Cells were treated with or without 200 nM MLi2, fixed with 2% PFA/PBS for 20 min at room temperature, and processed for immunocytochemistry as described below.

Cortical neural progenitor cells (NPCs) were generated according to the Shi protocol (Shi et al., 2012). Briefly, iPSCs (2 wells of a 6-well plate in E8 medium at 70–90% confluency) were replated into a geltrex pre-coated well of a 6-well plate in E8 medium containing 10 μM ROCK inhibitor to obtain a confluent cell monolayer the following day. Media was replaced with Neuronal Induction Media (NIM), consisting of Neural Maintenance Media (NMM) (50% DMEM:F12+glutamax (Thermofisher, 31331028), 50 % Neurobasal (Thermofisher, 12348017), 1 mM L-glutamine (Thermofisher, 25030024), 1 % B27 (Thermofisher, 17504044), 0.5% N2 (Thermofisher, 17502048), 0.05 mM 2-mercaptoethanol (Thermofisher, 31350), 0.5% non-essential amino acids (Thermofisher, 11140), 0.25 μg/mL insulin (Sigma, I9278)), 10 μM SB431542 (Tocris, 1614) and 1 μM dorsomorphine (Tocris, 3093). Media was replaced daily for the following 12 days. On day 12, after adding NIM, cells were passaged by incubation with 1.5 units/ml of dispase (ThermoFisher, 17105041) at 37°C for 3–10 min. Cell patches were gently collected, and washed twice with new NIM by letting patches settle in the bottom of a Falcon tube. Patches were gently resuspended in NIM and plated into 1% geltrex pre-coated wells at a split ratio of 1:3. The following day, media was replaced with NMM and 20 ng/mL bFGF (R&D Systems, 4114-TC). After four days of daily exchange of NMM/bFGF media, cells were split with dispase. Rosettes were plated into coverslips and processed for immunocytochemistry. At day 21, cells were split with dispase and then accutase, and NPCs were plated at 50′000 cells/ml in NMM containing 10 μM ROCK inhibitor, and fixed with 2% PFA/PBS for 20 min at room temperature either 2 h or 4 h after plating.

#### Cell culture and transfections

HEK293T cells were cultured in full medium consisting of DMEM with low glucose and pyruvate (ThermoFisher 11564446), 10% fetal bovine serum (ThermoFisher 11550356), non-essential amino acids (Sigma M7145) and 100 U/ml penicillin and 100 μg/mL streptomycin (ThermoFisher 11548876). Cells were transfected at 80% confluence with 6 μL of LipoD293 (Signagen SL100668) and 2 μg of GFP-tagged LRRK2 constructs (and 200 ng of Rab constructs, 300 ng of GAP constructs or 500 ng of myc-tagged rootletin, wildtype or constitutively active PP1α, or dominant-negative Nek2a constructs, respectively) per well of a 6-well plate for 5 h in full medium. The following day (80–90% confluence), cells were split to 25% confluence and processed for immunocytochemistry or Western blot analysis 48 h after transfection.

HeLa cells were cultured in full medium consisting of DMEM containing high glucose (ThermoFisher 11574486), 10% fetal bovine serum (Gibco 10500-064), non-essential amino acids (Sigma M7145), 1 mM sodium pyruvate (ThermoFisher 12539059) and 100 U/ml penicillin and 100 μg/mL streptomycin (ThermoFisher 11548876) at 37°C in 5% CO_2_. Confluent cells were rinsed with PBS and harvested by using 0.05% trypsin and 0.02 mM EDTA in PBS and subcultured at a ratio of 1:4–1:6. Cells were transfected at 80% confluence with 200 ng of GFP-tagged Rab8a constructs and 1 μL of Lipofectamine 2000 (Invitrogen™ 11668027) per well of a 12-well plate according to manufacturer's instructions for 5 h in DMEM. The following day, cells were split 1:3 onto coverslips, and were processed for immunocytochemistry 48 h after transfection.

A549 cells were cultured in DMEM containing high glucose without glutamine (Life Technologies 11960-044) and supplemented with 10% fetal bovine serum (Life Technologies 10500-064), 2 mM L-glutamine (Life Technologies 25030-024), 100 U/ml of Penicillin and 100 μg/mL of Streptomycin (Life Technologies 15140-122), and were subcultured at a ratio of 1:6-1:10 twice a week. Cells were transfected at 90% confluence with 1 μg of LRRK2 constructs (and 500 ng of myc-CDK5RAP2 or 1 μg of myc-tagged rootletin, wildtype or constitutively active PP1α, or dominant-negative Nek2a constructs, respectively) and 4 μL of LipoD293™ Transfection Reagent (SignaGen Laboratories SL100668), and 5 h later, media was replaced with full medium. Cells were split 1:4 onto coverslips the following day, and processed for immunocytochemistry or Western blotting 48 h after transfection.

Littermate matched wildtype and homozygous LRRK2-R1441C knockin MEFs generated from mice at E12.5 (resulting from crosses between heterozygous LRRK2-R1441C/wt mice maintained on a C57BL/6J background) were spontaneously immortalized by prolonged passaging, and were a generous gift from Prof. Dario Alessi (University of Dundee, UK) (Steger et al., 2017). Cells were grown in full medium consisting of DMEM containing high glucose (ThermoFisher 11960069), 10% fetal bovine serum (Gemini BioProducts, 900-208), 1 mM sodium pyruvate (ThermoFisher 11360070), non-essential amino acids (ThermoFisher 11140050), 2 mM L-glutamine (ThermoFisher 25030081), 100 U/ml penicillin and 100 μg/mL streptomycin (ThermoFisher 15140122). Cells were passaged at around 90% confluence to a ratio of 1:10 for general maintenance, with media exchanged every 2 days.

Primary human skin fibroblasts established from skin biopsies taken from three age- and sex-matched healthy control and three G2019S-LRRK2 PD patients, with informed consent and ethical approval, were grown in IMDM (ThermoFisher, 11510596) and 10% fetal bovine serum (ThermoFisher, 11560636), with media exchanged every two days. Cells were subcultured at a ratio of 1:2, and seeded at equal densities. All analyses were carried out on passages 7–12, and no passage-dependent differences were observed. Where indicated, cells were treated with MLi2, BI-2536, SB203580, SB202474 or SB202190.

### Methods details

#### DNA constructs and site-directed mutagenesis

GFP-tagged LRRK2 constructs, FLAG-tagged LRRK2 constructs, and human GFP-tagged Rab8a or mRFP-tagged Rab8a constructs have been previously published (Lara Ordonez et al., 2019; Madero-Pérez et al., 2018). The Rab8a-Q67L-T72A construct was generated by site-directed mutagenesis (QuikChange, Stratagene), and identity of constructs verified by sequencing of the entire coding region. Human GFP-tagged TBC1D30, TBC1D4/AS160, TBC1D4-R973K (catalytically inactive point mutant), TBC1D17 and TBC1D17-R381A (catalytically inactive point mutant) were a generous gift from Prof. Francis Barr (University of Oxford, UK) (Yoshimura et al., 2007). Myc-rootletin, myc-Nek2a, myc-Nek2a-K37R (kinase dead, dominant-negative) and myc-CDK5RAP2 were a generous gift from Prof. Erich Nigg (University of Basel, Switzerland), and myc-PP1α and myc-PP1α-T320A (catalytically active point mutant) from Prof. Len Neckers (NIH, USA), respectively. DNA was prepared from bacterial cultures using a midiprep kit (Promega) according to manufacturer's instructions.

#### Flow cytometry assays

For cell cycle analysis, HEK293T cells were transfected with GFP-tagged LRRK2 constructs and processed 48 h after transfection. Approximately 1 × 10^6^ cells were resuspended in PBS and fixed with 70% EtOH at −20°C for 10 min. Cells were washed twice with PBS, and the pellet resuspended and incubated in 340 μL PBS containing 100 μg/mL RNAse A (Roche, 10109169001) and 35 μg/mL propidium iodide (Sigma-Aldrich, P4864) for 30 min at 37°C in the dark. DNA content was analysed by flow cytometry on a FACScalibur (BD Biosciences), and data displayed using FlowJo software (Tree Star).

To analyse the percentage of cells in M phase, 2 × 10^6^ cells were fixed with 2% PFA/PBS for 15 min on ice. Cells were washed in PBS and incubated with an Alexa647-conjugated rabbit anti-phosphohistone H3 antibody (1:100; Cell Signaling, 9716) for 1 h at room temperatue in 10% goat serum (Vector Laboratories, 5–1000). Cells were washed in PBS and analyzed using flow cytometry as described above.

MEF cells were seeded into T25 flasks and collected at 90–100% confluency. Cells were pelleted at 500 x g for 5 min, and the pellet washed twice with PBS (ThermoFisher, 10010-023). After the second wash, the cell pellet (containing 20–50 μL PBS) was gently tapped to make a cell suspension. Cells were fixed with ice-cold 70% EtOH which was added dropwise while the cells were gently vortexed, and fixed cells were kept at −20°C for 48 h. Fixed cells were centrifuged at 500 x g for 5 min and washed twice with PBS to remove EtOH, followed by one wash with stain buffer (BD Biosciences, 554657). To determine the percentage of cells in M phase, cells were incubated with 20 μL of rat anti-phosphohistone H3 (pS28) antibody (BD Biosciences, 558217) in 80 μL of stain buffer for 20 min at room temperature in the dark. Cells were washed twice with stain buffer, and incubated with 500 μL of propidium iodide/RNase solution (BD Biosciences, 550825) for 15 min at room temperature in the dark, followed by cell cycle analysis in a BD LSRII flow cytometer. Data analysis was performed using ModFit LT software, and the percentage of mitotic cells determined by FlowJo software.

#### Immunofluorescence and laser confocal imaging

Cells were fixed with 4% PFA/PBS (HEK293T and A549) or with 2% PFA/PBS (iPSCs and NPCs) for 20 min at room temperature, followed by ice-cold MeOH for 2 min when employing anti-γ-tubulin antibody. Primary human dermal fibroblasts were fixed with 4% PFA/PBS for 20 min at 37°C, followed by ice-cold MeOH at −20°C for 5 min. Cells were permeabilized with 0.2% Triton-X100/PBS for 10 min at room temperature, followed by incubation in blocking solution (0.5% BSA (w/v) in 0.2% Triton-X100/PBS) for 1 h at room temperature. MEF cells were fixed with ice-cold MeOH at −20°C for 10 min, followed by permeabilization in ice-cold 0.1% Triton-X100/PBS for 5 min, and coverslips blocked with 1% BSA in 0.1% Triton-X100/PBS for 1 h at room temperature. Primary antibodies were diluted in blocking solution and incubated overnight at 4°C. Primary antibodies included rabbit polyclonal anti-pericentrin (Abcam, ab4448, 1:1000), mouse monoclonal anti-pericentrin (Abcam, ab28144, 1:1000), mouse monoclonal anti-γ-tubulin (Abcam, ab11316, 1:1000), mouse monoclonal anti-FLAG (Sigma, clone M2, F1804, 1:1000), mouse monoclonal anti-c-myc (Sigma, clone 9E10, M4439, 1:500), rabbit monoclonal anti-LRRK2 (Abcam, ab133518, 1:1000), rabbit polyclonal anti-Arl13b (Proteintech, 17711-1-AP, 1:250), rabbit monoclonal anti-phospho-T73-Rab10 (Abcam, ab241060, 1:100), mouse monoclonal anti-nestin (Novus, mab1259, 1:50), rabbit polyclonal anti-Pax6 (Biolegend, PRB-278P, 1:300), mouse monoclonal anti-Ki67 (BD Biosciences, 550609, 1:100), mouse monoclonal anti-rootletin (Santa Cruz Biotechnology, sc-374056, 1:500), rabbit polyclonal anti-cep68 (Proteintech, 15147-1-AP, 1:500), rabbit polyclonal anti-CDK5RAP2 (Bethyl, A300-554A, 1:500), mouse monoclonal anti-CDK5RAP2 (Sigma, AMAB91163, 1:500), rabbit polyclonal anti-CDK5RAP2 (Sigma, 06-1398, 1:2500; for MEF cells only), rabbit polyclonal anti-cNAP1 (Proteintech, 14498-1-AP, 1:500), rabbit polyclonal anti-ninein (Abcam, ab4447, 1:500), rabbit polyclonal anti-cenexin (Abcam, ab43840, 1:500) and mouse monoclonal anti-cyclinB1 (Santa Cruz Biotechnology, sc-245, 1:200). Rabbit polyclonal anti-rootletin was a generous gift from Prof. Erich Nigg. The next day, coverslips were washed three times 10 min in 0.2% Triton-X100/PBS (wash buffer), and then incubated with secondary antibodies diluted in wash buffer for 1 h at room temperature. Secondary antibodies included Alexa405-conjugated goat anti-mouse or goat anti-rabbit, Alexa488-conjugated goat anti-mouse or goat anti-rabbit, and Alexa594-conjugated goat anti-mouse or goat anti-rabbit, Alexa555-conjugated goat anti-mouse or goat anti-rabbit, and Alexa647-conjugated goat anti-mouse or goat anti-rabbit (all from Invitrogen, 1:1000). Coverslips were washed two times in wash buffer, once in PBS, and then mounted in mounting medium with DAPI (Vector Laboratories).

For determination of the subcellular localization of GFP-tagged Rab10 proteins, HeLa cells were fixed with 4 % PFA in PBS for 20 min at room temperature, coverslips were washed with PBS and mounted in mounting medium with DAPI (Vector Laboratories).

#### Proximity ligation assays

Proximity ligation assays (PLAs) were performed 48 h after transient transfection using DuoLink PLA Technology according to manufacturer’s instructions (Sigma-Aldrich; Duolink *In Situ* PLA probe anti-rabbit PLUS (DUO92002), Duolink *In Situ* PLA probe anti-mouse MINUS (DUO92004), Duolink *In Situ* Detection Reagents Red (DUO92008). Upon fixation of cells in 2% PFA/PBS for 20 min at room temperature, cells were washed 3 × 10 min in PBS, and coverslips blocked in 1 x PLA blocking solution (provided in kit) for 1 h at 37°C, followed by incubation with primary antibodies overnight at 4°C using PLA antibody diluent (provided in kit). Primary antibodies included rabbit polyclonal anti-pericentrin (Abcam, ab4448, 1:500) and mouse monoclonal anti-cdk5rap2 (Sigma, AMAB91163, 1:500). All incubations were performed in the dark.

#### Image acquisition and quantification

Images were acquired on a Leica TCS-SP5 confocal microscope using a 63×1.4 NA oil UV objective (HCX PLAPO CS). Images were collected using single excitation for each wavelength separately and dependent on secondary antibodies (488 nm argon laser line and a 510–540 nm emission bandpass (for Alexa488), 561 nm HeNe laser line and a 596–628 nm emission band pass (for Alexa594), 633 nm HeNe laser line and and a 660–705 nm emission band pass (for Alexa647), and 561 nm HeNe laser line and a 587–623 nm emission band pass (for Alexa555)). GFP-tagged proteins were excited with a 488 nm argon laser line and 506–545 a nm emission band pass, mRFP-tagged proteins with a 561 nm HeNe laser line and a 596–628 nm emission band pass, and DAPI with a 405 nm UV diode and a 420–470 nm emission band pass, respectively. Alternatively, images were acquired on an Olympus FV1000 Fluoview confocal microscope using a 60×1.2 NA UPlanSApo water objective.

For centrosome cohesion determination, 15–20 image sections of selected areas were acquired with a step size of 0.5 μm, and z-stack images analyzed and processed using Leica Applied Systems (LAS AF6000) image acquisition software, FV10-ASW4.1 software (Olympus) or employing Fiji. The same laser intensity settings and exposure times were used for image acquisition of individual experiments to be quantified. The average distance between duplicated centrosomes is cell type-dependent. Original studies in U2OS cells determined the distance between duplicated centrosomes to be < 2 μm in 90–95% of cells, and centrosome splitting was defined as cells where the duplicated centrosomes were >2 μm apart (Meraldi and Nigg, 2001). Performing identical analyses, centrosomes were scored as separated when the distance between their centers was >1.5 μm (HEK293T, iPS and NPC), >2.5 μm (A549, primary human dermal fibroblasts), or >5 μm (MEF cells), respectively. In all cases, mitotic cells were excluded from the analysis. For most experiments, images were analyzed by one or two additional observers blind to condition, with identical results obtained in all cases.

Integrated density determinations were performed over non-saturated images acquired with the same acquisition settings. A circle with diameter of 1.6 μm was placed over each centrosome as identified by pericentrin staining, and integrated density of centrosomal staining determined for each cell, with background intensity adjacent to the centrosome subtracted in all cases. An average of 50–70 individual cells per condition per experiment were quantified in this manner.

For determination of the percentage of ciliated cells, around 200 random cells per experiment were scored for either the absence or presence of primary cilia as assessed using Arl13b staining. Quantification was performed by two observers blind to condition, with identical results obtained in both cases. For determination of proximity ligation assays (PLA), 100–120 random non-transfected or transfected cells were scored for the presence or absence of a perinuclear dot-like PLA signal per condition and experiment by an observer blind to condition.

#### Cell extracts and Western blotting

Cells were collected 48 h after transfection from a well of a 6-well plate, washed in PBS, followed by resuspension in 75 μL of PBS and lysis with 25 μL of 5x SDS sample buffer containing 2.5% (v/v) β-mercapthoethanol, and boiling for 5 min at 95°C. Around 15–20 μL (around 40 μg of protein) were resolved by SDS-PAGE polyacrylamide gel electrophoresis using 4–20% precast gradient gels (Bio-Rad, 456–1096), and proteins were electrophoretically transferred onto nitrocellulose membranes (GE Healthcare). Membranes were blocked in blocking buffer (Li-COR Biosciences, Li-COR Odyssey Intercept blocking buffer, 927–70001) or with 0.1% Tween-20/TBS and 5% (w/v) BSA for 1 h at room temperature, and incubated with primary antibodies in blocking buffer overnight at 4°C. Primary antibodies included rabbit polyclonal anti-GFP (Abcam, ab6556, 1:1000), mouse monoclonal anti-FLAG (Sigma, clone M2, F1894, 1:1000), mouse monoclonal anti-c-myc (Sigma, clone 9E10, M4439, 1:1000), mouse monoclonal anti-α-tubulin (Sigma, clone DM1A, T6199, 1:10′000), mouse monoclonal anti-GAPDH (Santa Cruz Biotechnology, sc-32233, 1:2000), rabbit monoclonal anti-phospho-S935-LRRK2 (Abcam, ab133450, 1:500), rabbit monoclonal knockout-validated anti-phospho-T72-Rab8a (Abcam, ab230260, 1:1000), mouse monoclonal anti-Rab8 (BD Biosciences, 610844, 1:1000), rabbit monoclonal anti-phospho-T73-Rab10 (Abcam, ab230261, 1:1000), mouse monoclonal knockout-validated anti-Rab10 (Sigma, SAB5300028, 1:1000), rabbit polyclonal anti-cep290 (Abcam, ab85728, 1:1000), rabbit polyclonal anti-cenexin (Abcam, ab43840, 1:500), rabbit polyclonal anti-IFT88 (Proteintech, 13967-1-AP, 1:1000), mouse monoclonal anti-γ-tubulin (Abcam, ab11316, 1:10000), rabbit polyclonal anti-CDK5RAP2 (Bethyl, A300-554A, 1:1000), rabbit polyclonal anti-CDK5RAP2 (Sigma, 06-1398, 1:2500; for MEF cells only), rabbit polyclonal anti-cep68 (Proteintech, 15147-1-AP, 1:1000), mouse monoclonal anti-LAMP1 (Santa Cruz, sc-20011, 1:1000) and mouse monoclonal anti-Rab11 (BD Biosciences, 610656, 1:1000). Secondary antibodies included goat anti-rabbit or anti-mouse IRDye 800CW, or goat anti-rabbit or anti-mouse IRDye 680RD (1:7000). Blots were imaged via near infrared fluorescent detection using Odyssey CLx Imaging System, and quantification was performed using the instrument's Image Studio Software.

#### Centrosome isolation

Centrosomes were isolated from adherent cells using hypotonic cell lysis and purification on sucrose gradients according to the Gogendeau protocol (Gogendeau et al., 2015). Briefly, exponentially growing non-transfected or transfected HEK293T cells (4 × 10^7^ cells in two T175 cell culture flasks) were incubated with culture medium containing 1 μg/mL nocodazole and 1 μg/mL cytochalasin D (Sigma, C2618) for 1 h at 37°C to depolymerize microtubule and actin filaments. Cells attached to flasks were gently washed with 10 mL ice-cold PBS, followed by a wash with 10 mL ice-cold 0.1 X PBS containing 8% sucrose, and a wash with 10 mL ice-cold hypotonic lysis buffer (1 mM PIPES pH 7.2, 0.75 mM MgCl_2_, 1% β-mercaptoethanol, 1 mM Na_3_VO_4_, 5 mM NaF, 1 mM PMSF and protease inhibitor cocktail (Roche, 4693124001). Cell lysis was performed with a total of 5 mL lysis buffer containing 0.5% NP-40 for 10 min at 4°C on a shaker. Lysates were cleared by centrifugation at 2′500 x g for 10 min at 4°C, and the supernatant was adjusted to a final 10 mM PIPES, pH 7.2 concentration, layered on top of 1 mL of a 60% sucrose cushion in gradient buffer (10 mM PIPES pH 7.2, 0.1% Triton-X100, 0.1% β-mercaptoethanol), and centrifuged at 10′000 x g for 40 min at 4°C. The supernatant was carefully removed, and around 30% of the initial volume collected from the bottom of the tube. This fraction, enriched for centrosomes, was diluted to around 8% sucrose in lysis buffer containing 10 mM PIPES, and loaded on top of a discontinuous sucrose gradient (2 mL 70% sucrose, 1 mL 50% sucrose, 1 mL 40% sucrose in gradient buffer) in ultracentrifuge tubes (Beckman Ultra-Clear, 14 × 95 mm, cat. no. 344060). Centrosomes were isolated by ultracentrifugation at 120′000 x g for 2 h at 4°C using an SW40Ti rotor (Beckman), and fractions collected from the bottom using a Beckman recovery system (1 mL for first fraction, and 0.3 mL for all subsequent fractions). Samples were resuspended in 5 x SDS sample buffer, and 20 μL of each fraction resolved by SDS-PAGE and Western blotting with the indicated antibodies.

### Quantification and statistical analysis

All the statistical details of the experiment can be found in the legend of each figure. For each statistical analysis, at least three independent experiments were carried out. Data were analyzed with a one-way ANOVA and Tukey's *post-hoc* test, and p < 0.05 was considered significant. The following significance thresholds were used throughout the study: ∗∗∗∗p < 0.001; ∗∗∗p < 0.005; ∗∗p < 0.01; ∗p < 0.05. The values in the bar and line graphs represent the means +/− s.e.m. in all figures.

## Data Availability

All immunoblotting and microscopy data reported in this paper will be shared by the lead contact upon request. This paper does not report original code. Any additional information required to reanalyze the data reported in this paper is available from the lead contact upon request.
