# Supplementary material for: Pathogenic LRRK2 regulates centrosome cohesion via Rab10/RILPL1-mediated CDK5RAP2 displacement
Source: iScience. 2022 May 30;25(6):104476. doi: 10.1016/j.isci.2022.104476 (PMC9198432; doi:10.1016/j.isci.2022.104476)
Supplement: Document S1. Figures S1–S12 and Table S1 [file mmc1.pdf]

iScience, Volume *25*

Supplemental information

Pathogenic LRRK2 regulates centrosome cohesion via Rab10/RILPL1-mediated CDK5RAP2 displacement

Elena Fdez, Jesús Madero-Pérez, Antonio J. Lara Ordóñez, Yahaira Naaldijk, Rachel Fasiczka, Ana Aiastui, Javier Ruiz-Martínez, Adolfo López de Munain, Sally A. Cowley, Richard Wade-Martins, and Sabine Hilfiker

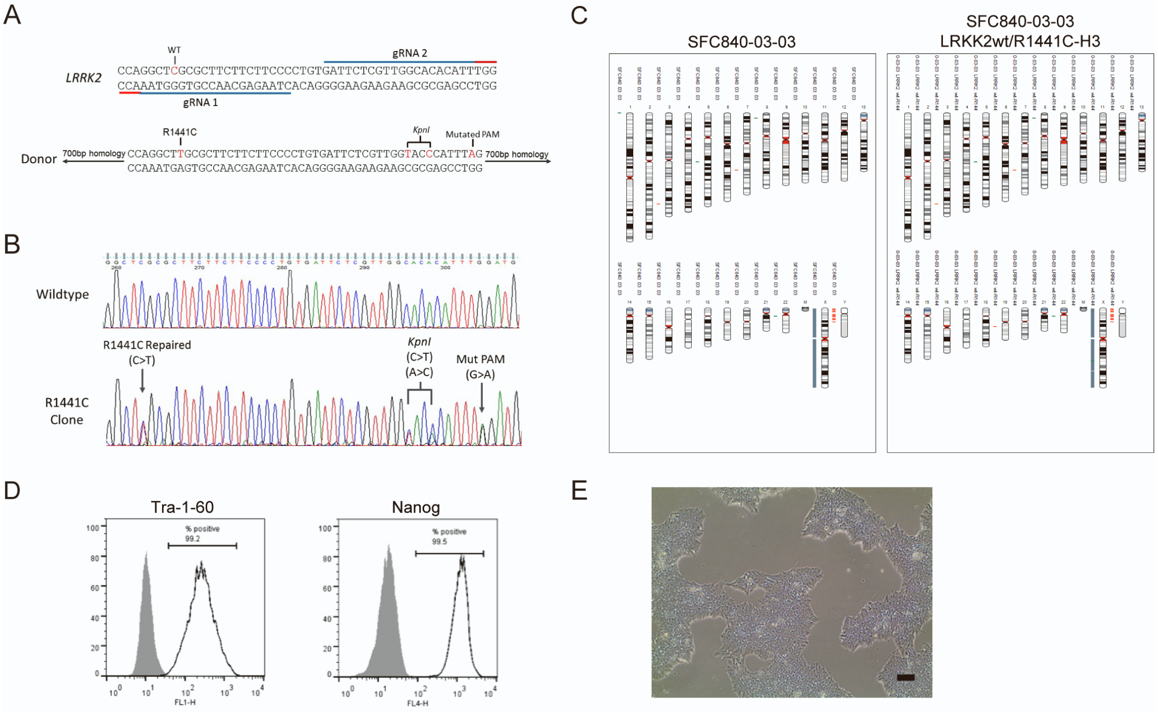

Figure S1. Generation and characterization of an isogenic LRRK2-R1441C iPSC line (SFC840-03-03 LRKK2wt/R1441C-H3). Related to Figure 1 and STAR Methods. (**A**) CRISPR/Cas-9 mediated engineering strategy using double nickase and homology directed repair with donor DNA template. gRNAs targeting the endogenous LRRK2 locus indicated with blue lines and protospacer adjacent motif [PAM] indicated in red lines. Donor DNA template with the R1441C mutation, a silent mutation in the PAM sequence (to increase gene-editing efficiency) and two silent mutations to introduce a *KpnI* restriction site to facilitate clone screening. (**B**) Sequencing to confirm the introduction of mutations as indicated in (A). (**C**) Genome integrity of parental SFC840-03-03 (left) and gene-edited SFC840-03-03 LRKK2wt/R1441C-H3 (right) iPSC lines assessed by Illumina Human OmniExpress24-v1-1 SNP array. Karyograms (KaryoStudio, Illumina) show amplifications (green)/deletions (orange)/LOH regions (grey) alongside the relevant chromosome (NB two X chromosomes annotated grey, Y chromosome orange). (**D**) Flow cytometry analysis confirming expression of pluripotency markers Tra-1-60 and Nanog. Open grey plot represents antibody control, filled white plot represents the SFC840-03-03 LRKK2wt/R1441C-H3 iPSC line. (**E**) Representative phase-contrast images of the SFC840-03-03 LRKK2wt/R1441C-H3 iPSC line 24h (left) and 72h (right) post-thaw showing typical colony morphology. Scale bar, 100 μm.

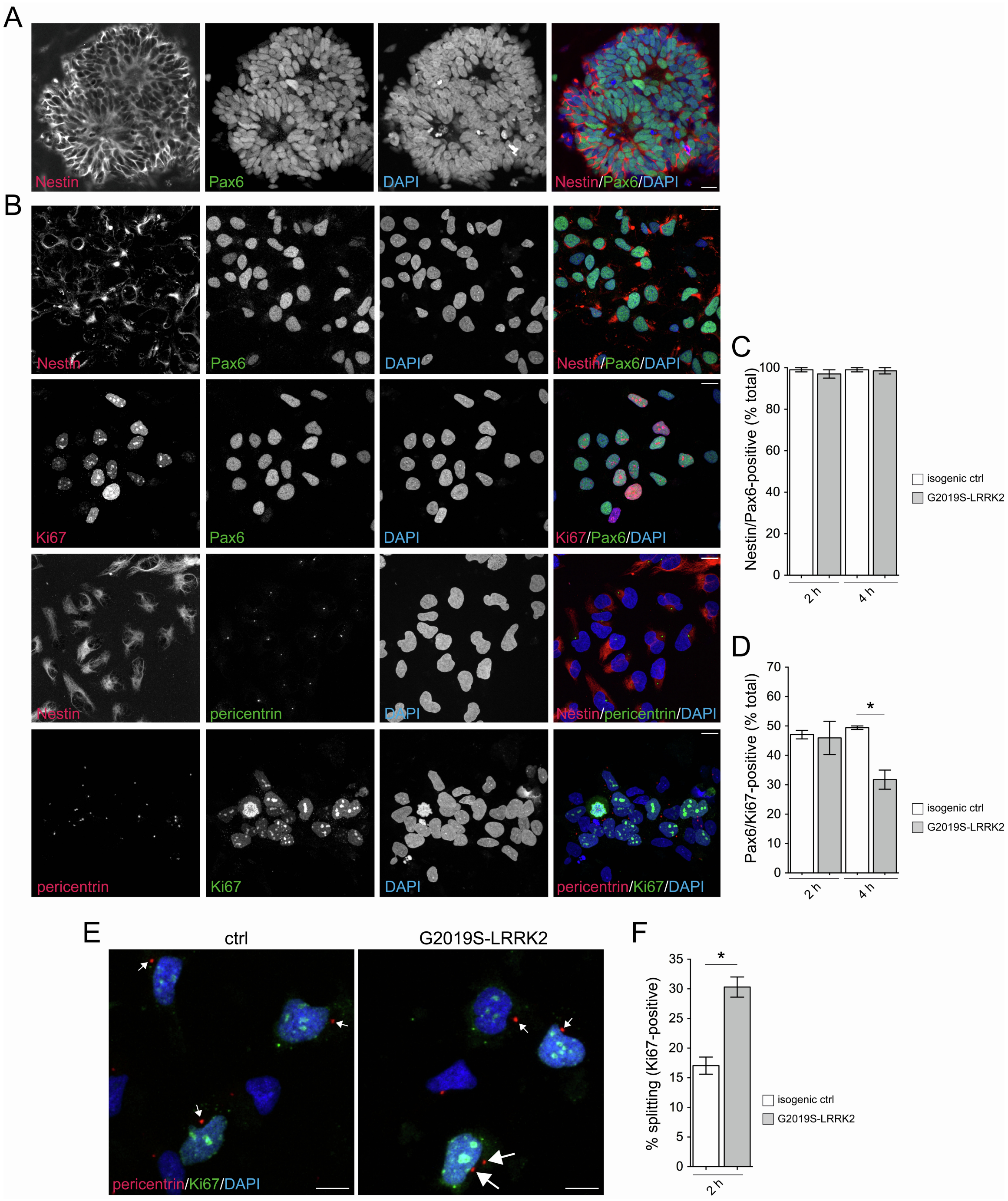

Figure S2. Pathogenic LRRK2 causes centrosomal cohesion deficits in cortical neural progenitor cells (NPCs). Related to Figure 1. A cortical differentiation protocol was employed to obtain NPCs from a LRRK2$^{G2019S/WT}$ (G2019S-LRRK2) Parkinson´s disease patient iPSC line and from the isogenic, gene-corrected LRRK2$^{WT/WT}$ (ctrl) line. (**A**) Neural rosettes from control line stained for neural progenitor markers nestin and pax6, with nuclei stained with DAPI. Scale bar, 20 μm. (**B**) At day 21, dissociated control NPCs were plated onto geltrex pre-coated coverslips, and 2 h later fixed and stained for

NPC markers nestin and pax6, proliferation marker Ki67, or centrosomal marker pericentrin as indicated, with DAPI in blue. Scale bar, 10 μm. (**C**) Quantification of both nestin- and pax6-positive NPCs from G2019S-LRRK2 or isogenic control line, fixed either 2 h or 4 h after plating. Around 150 cells per condition and experiment were quantified. Bars represent mean ± s.e.m. (n=2 independent experiments). (**D**) Quantification of both pax6- and Ki67-positive NPCs from G2019S-LRRK2 or isogenic control line, fixed either 2 h or 4 h after plating. A significant decrease in proliferative capacity was observed in G2019S-LRRK2 as compared to gene-corrected control line 4 h after plating. Around 150 cells per condition and experiment were quantified. Bars represent mean ± s.e.m. (n=2 independent experiments; isogenic ctrl versus G2019S-LRRK2, $p = 0.033$); $*p < 0.05$. (**E**) Example of isogenic control or G2019S-LRRK2 NPC cells, plated onto geltrex pre-coated coverslips, fixed 2 h after plating, and stained for proliferative marker Ki67, centrosomal marker pericentrin and DAPI. Arrows point to centrosomes in proliferative cells. Scale bar, 10 μm. (**F**) Quantification of the percentage of control or G2019S-LRRK2 NPCs with split centrosomes (duplicated centrosomes with a distance between their centers > 1.5 μm). Around 150 Ki67-positive cells were quantified per condition and experiment. Bars represent mean ± s.e.m. (n=2 independent experiments; isogenic ctrl versus G2019S-LRRK2, $p = 0.027$); $*p < 0.05$.

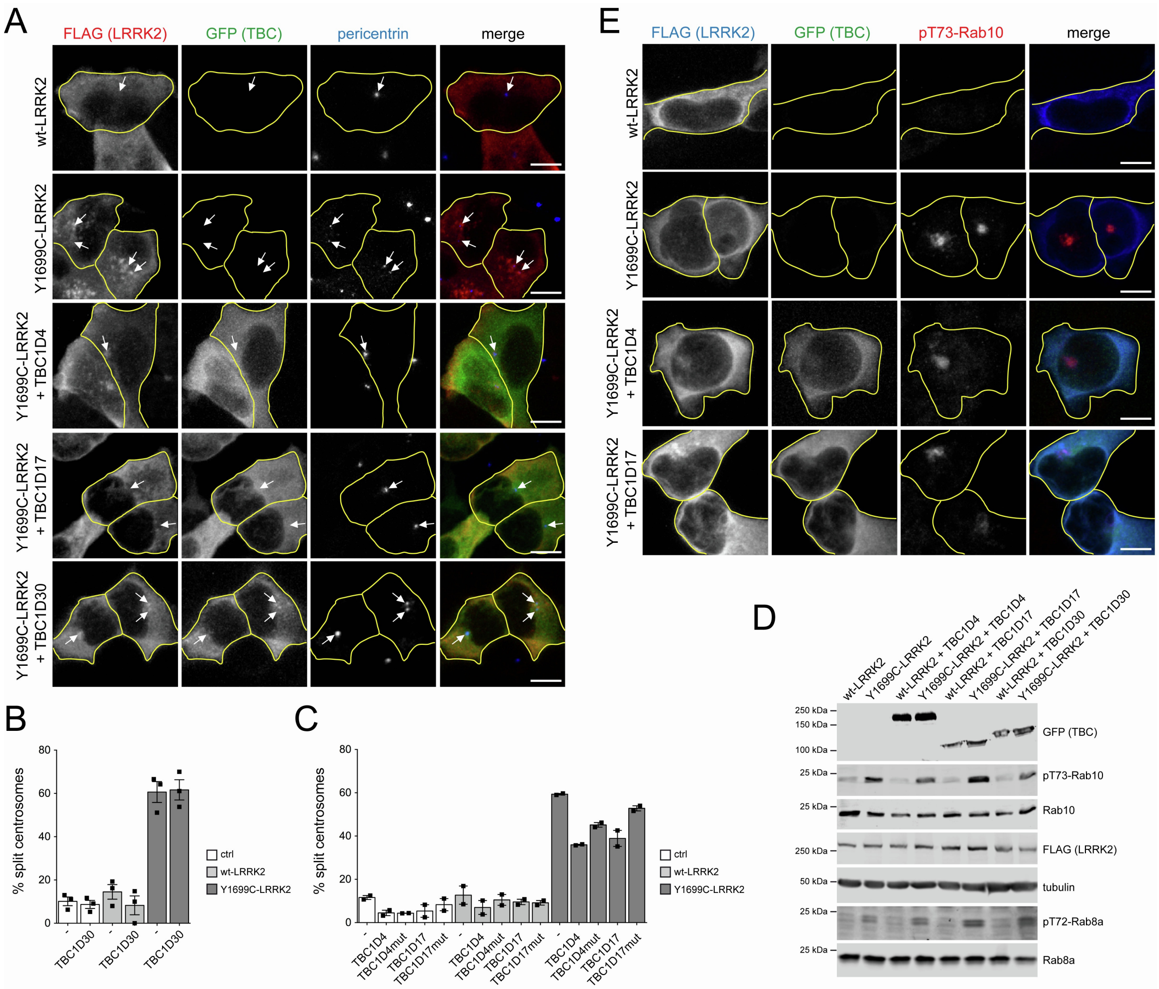

Figure S3. GAPs for Rab8a or Rab10 are unable to modify LRRK2-mediated centrosomal cohesion deficits. Related to Figure 2. (**A**) Example of HEK293T cells transfected with FLAG-tagged wildtype or Y1699C-mutant LRRK2 along with GFP-tagged TBC1D4, TBC1D17 or TBC1D30 as indicated, and stained with pericentrin. Arrows point to centrosomes in transfected cells. Cell boundaries (yellow) are shown and were determined by FLAG staining due to FLAG-tagged LRRK2 expression. Scale bar, 10 μm. (**B**) Quantification of the split centrosome phenotype in either non-transfected cells (ctrl), or in cells transfected with wildtype or Y1699C-mutant LRRK2, in either the presence or absence of GFP-tagged TBC1D30 co-transfection as indicated. Bars represent mean ± s.e.m. (n=3 independent experiments). (**C**) As in (B), but cells co-transfected with GFP-tagged TBC1D17, TBC1D4, or inactive point-mutated versions thereof. Bars represent mean ± s.e.m. (n=2 independent experiments). (**D**) Cells were transfected with either FLAG-tagged wildtype or Y1699C-mutant LRRK2, and co-transfected with different GFP-tagged TBC constructs as indicated, and extracts blotted for GFP-tagged TBC constructs, FLAG-tagged LRRK2, pT72-Rab8a, pT73-Rab10, total Rab8a, total Rab10 or tubulin as loading control. (**E**) Example of cells transfected with FLAG-tagged wildtype or Y1699C-mutant LRRK2, or FLAG-tagged Y1699C-LRRK2 along with either GFP-tagged TBC1D4 or TBC1D17 as indicated, and stained with pT73-Rab10 antibody. Cell boundaries (yellow) are shown and were determined by

FLAG staining due to FLAG-tagged LRRK2 expression. Scale bar, 10 μm. Transfection efficiencies were 20 ± 4 % (n=3) with no significant differences between constructs observed. Co-transfection efficiencies were around 85-90 % in all cases, with no differences between conditions (wt + TBC1D4 = 93 ± 3 %; Y1699C + TBC1D4 = 87 ± 1.7 %; wt + TBC1D17 = 82 ± 7 %; Y1699C + TBC1D17 = 83 ± 4 %; wt + TBC1D30 = 90 ± 4 %; Y1699C + TBC1D30 = 87 ± 6 %; n=3 independent experiments).

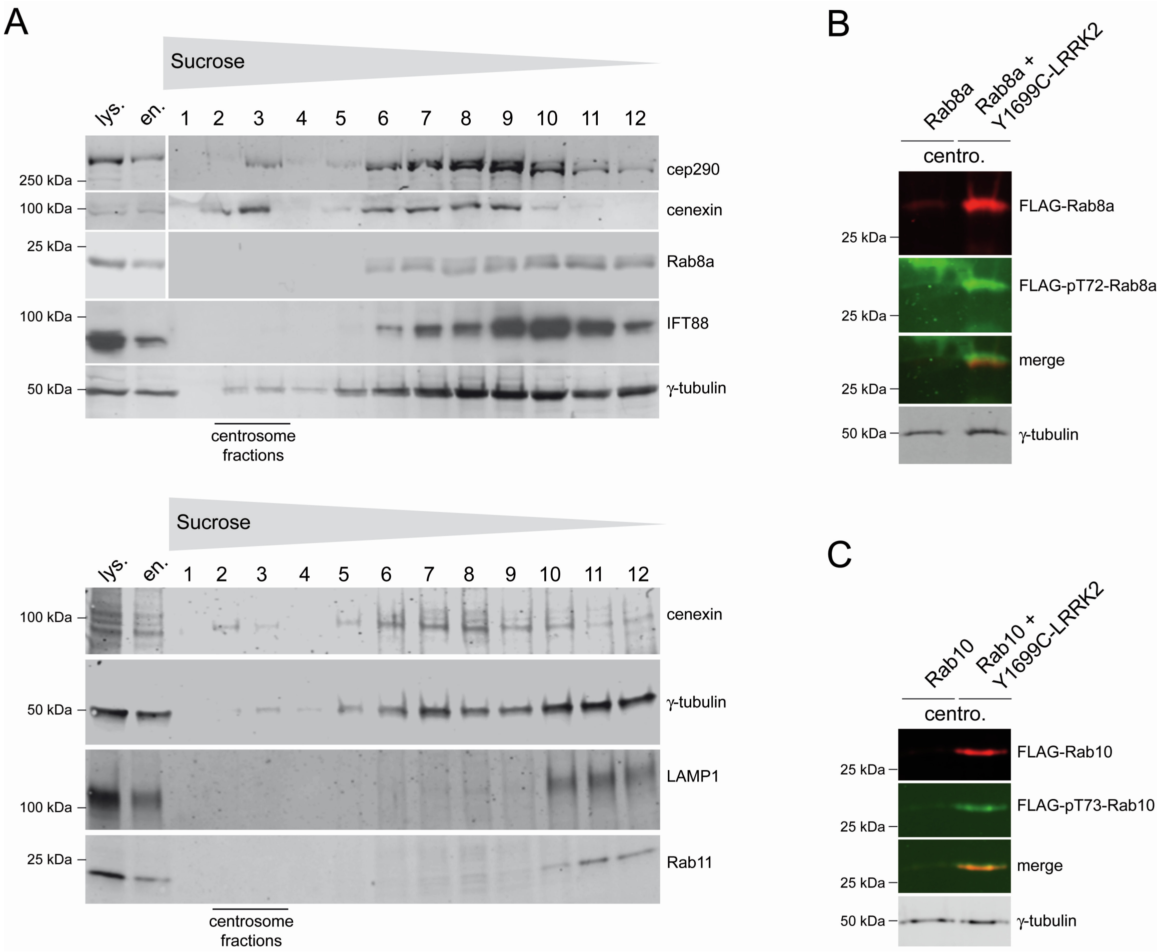

Figure S4. Phospho-Rab8a and phospho-Rab10 accumulate on centrosomes in the presence of pathogenic LRRK2 expression. Related to Figure 3. (**A**) HEK293T cells were lysed, centrosomes enriched over a 60 % sucrose cushion, followed by purification over a discontinuous sucrose gradient as described in Materials and Methods. The lysate (lys.), enriched fraction (en.) and sucrose fractions were resolved by SDS-PAGE and Western blotting with the indicated antibodies, including various centrosomal markers and Rab8a (top) or markers for endosomes (Rab11) or endolysosomes (LAMP1) (bottom). Centrosomes were recovered in around 50 % sucrose fractions. (**B**) HEK293T cells were transfected with either FLAG-tagged Rab8a or with FLAG-tagged Rab8a and GFP-tagged Y1699C-mutant LRRK2 as indicated, and centrosome fractions analyzed for levels of FLAG-tagged Rab8a (FLAG-Rab8a), FLAG-tagged pT72-Rab8a (FLAG-pT72A-Rab8a) or γ-tubulin as indicated. (**C**) HEK293T cells were transfected with either FLAG-tagged Rab10 or with FLAG-tagged Rab10 and GFP-tagged Y1699C-mutant LRRK2, and centrosome fractions analyzed for levels of FLAG-tagged Rab10 (FLAG-Rab10), FLAG-tagged pT73-Rab10 (FLAG-pT73-Rab10) or γ-tubulin as indicated.

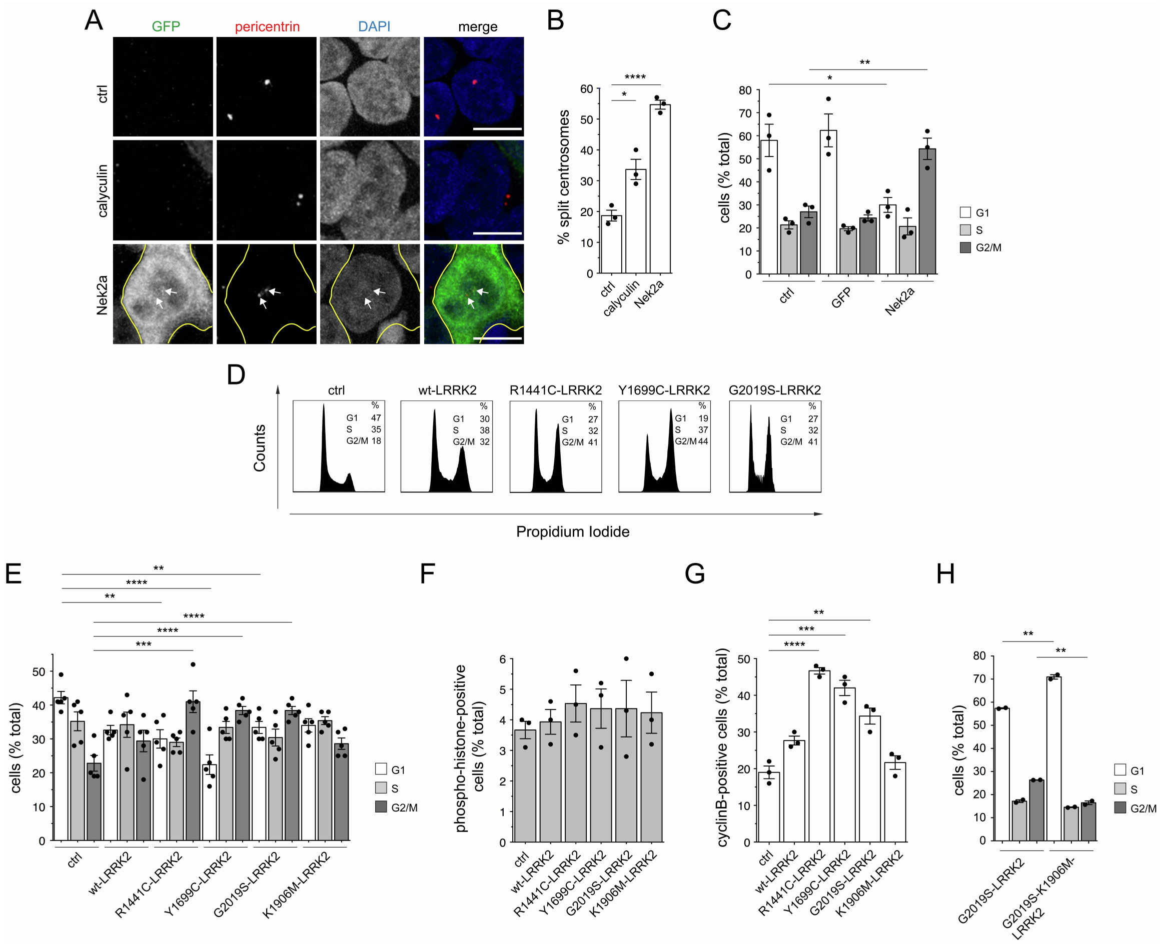

Figure S5. Centrosomal cohesion deficits triggered by Nek2a or LRRK2 expression cause cell cycle alterations. Related to Figure 3. (**A**) Example of HEK293T control cells, cells treated with calyculin A (10 nM, 1 h) or co-expressing GFP and myc-tagged Nek2a, and stained with pericentrin antibody and DAPI. Arrows point to centrosomes in transfected cells. Cell boundaries (yellow) are shown and were determined by GFP due to GFP co-expression. Scale bar, 10 μm. (**B**) Quantification of the type of experiments depicted in (A) indicate that calyculin A or expression of Nek2a cause premature centrosome splitting. An average of 30 cells with two centrosomes were analyzed for each condition. Bars represent mean ± s.e.m. (n=3 independent experiments; ctrl versus calyculin, $p = 0.015$; ctrl versus Nek2a, $p < 0.001$); ****$p < 0.001$; *$p < 0.05$. (**C**) The percentage of cells in each phase of the cell cycle was quantified by FACS from non-transfected cells, cells transfected with GFP, or cells co-transfected with GFP and Nek2a. Bars represent mean ± s.e.m. (n=3 independent experiments; G1: ctrl versus Nek2a, $p = 0.022$; G2/M: ctrl versus Nek2a, $p = 0.006$); **$p < 0.01$; *$p < 0.05$. (**D**) Propidium iodide-stained nuclei of control, or of cells expressing GFP-tagged LRRK2 constructs as indicated, were quantified by FACS as a percentage of total cells analyzed. The percentage of cells in each phase of the cell cycle is indicated to the right of each histogram. (**E**) Quantification of experiments of the type depicted in (D). Bars represent mean ± s.e.m. (n=5 independent experiments; G1: ctrl versus R1441C-LRRK2, p =

0.005; ctrl versus Y1699C-LRRK2, $p < 0.001$; ctrl versus G2019S-LRRK2, $p = 0.008$; G2/M: ctrl versus R1441C-LRRK2, $p = 0.001$; ctrl versus Y1699C-LRRK2, $p < 0.001$; ctrl versus G2019S-LRRK2, $p < 0.001$); ****$p < 0.001$; ***$p < 0.005$; **$p < 0.01$. (**F**) Transfected cells were stained with a fluorescently-labelled anti-phospho-histone H3 antibody and analyzed using FACS. Bars represent mean ± s.e.m. (n=3 independent experiments). (**G**) Cells were transfected with the indicated GFP-tagged LRRK2 constructs, and the percentage of cells displaying an accumulation of cytosolic cyclin B1 scored from 50-100 cells per condition. Bars represent mean ± s.e.m. (n=3 independent experiments; ctrl versus R1441C-LRRK2, $p < 0.001$; ctrl versus Y1699C-LRRK2, $p = 0.001$; ctrl versus G2019S-LRRK2, $p = 0.005$); ****$p < 0.001$; ***$p < 0.005$; **$p < 0.01$. (**H**) The percentage of cells in each phase of the cell cycle was quantified by FACS from cells transfected with either G2019S-LRRK2 or kinase-inactive G2019S-K1906M-LRRK2 mutant. Bars represent mean ± s.e.m. (n=2 independent experiments; G1: G2019S versus G2019S-K1906M, $p = 0.005$; G2/M: G2019S versus G2019S-K1906M, $p = 0.006$); **$p < 0.01$.

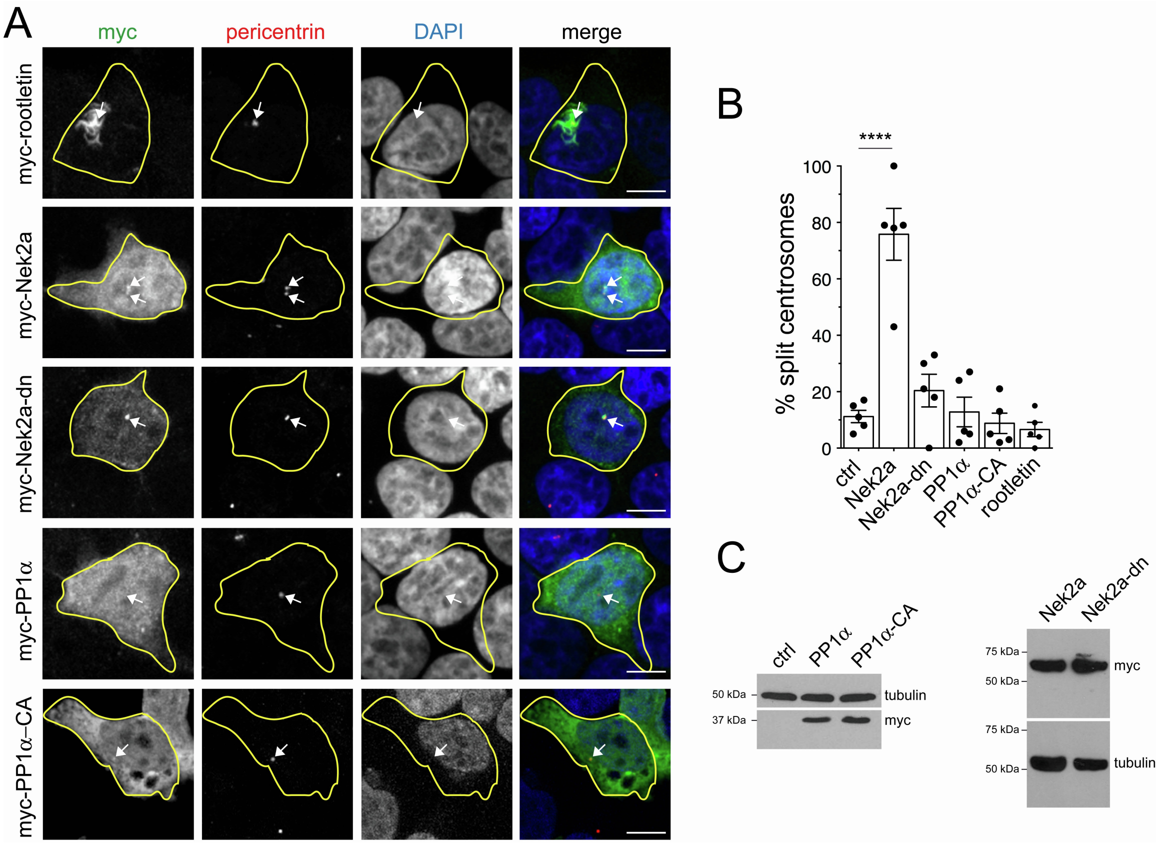

Figure S6. Expression of rootletin, dominant-negative Nek2a or PP1α have no effect on centrosomal cohesion. Related to Figure 3. (**A**) Example of HEK293T cells transfected with the indicated myc-tagged constructs, and stained with pericentrin antibody and DAPI. Arrows point to centrosomes in transfected cells. Cell boundaries (yellow) are shown and were determined by myc staining due to myc-tagged construct expression. Scale bar, 10 μm. (**B**) Quantification of the split centrosome phenotype from experiments of the type depicted in (A). Bars represent mean ± s.e.m. (n=5 independent experiments; ctrl versus Nek2a, $p < 0.001$); ****$p < 0.001$. (**C**) Cells were transfected with myc-tagged PP1α constructs, or with myc-tagged Nek2a constructs as indicated, and extracts blotted with anti-myc antibody and tubulin as loading control.

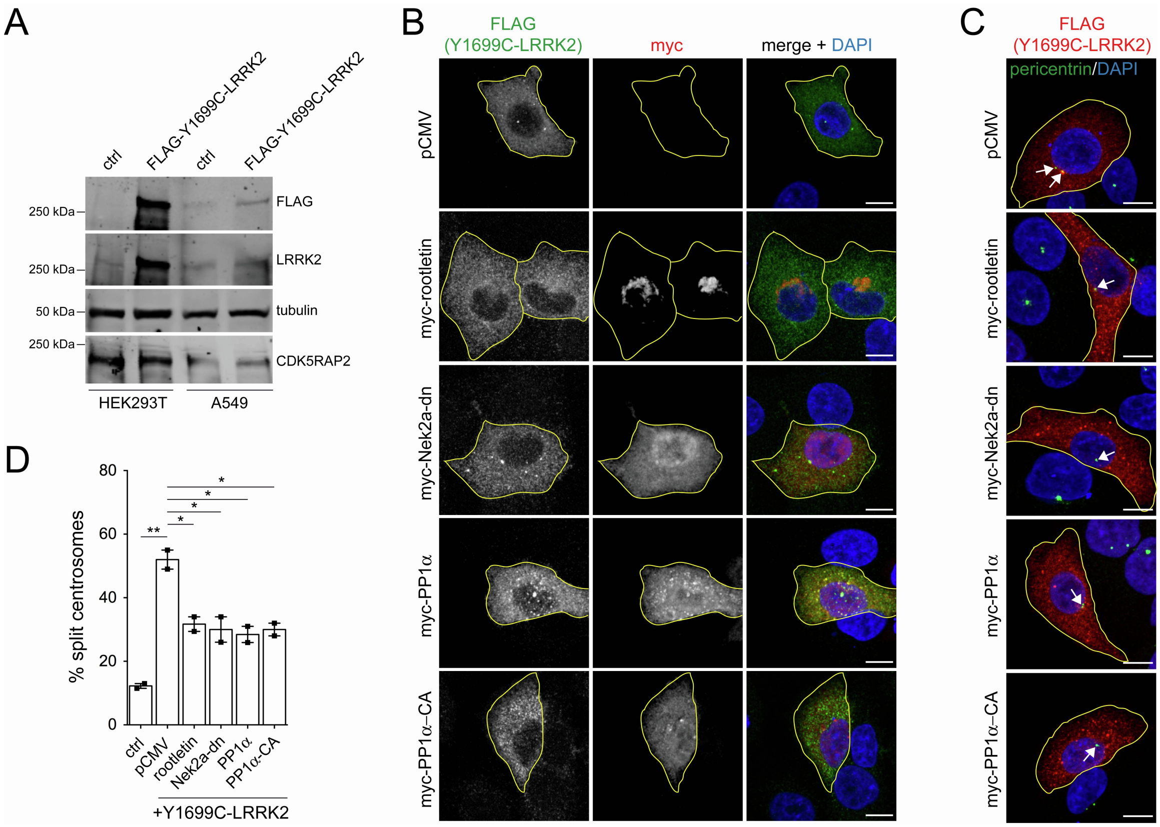

Figure S7. Rescue of LRRK2-mediated cohesion deficits by expression of centrosomal linker protein rootletin or by Nek2a/PP1α modulation in A549 cells. Related to Figure 3. (**A**) HEK293T or A549 cells were transfected with either pCMV (ctrl) or FLAG-tagged Y1699C-LRRK2, and extracts blotted for FLAG, LRRK2, CDK5RAP2 or tubulin as loading control. (**B**) Example of A549 cells co-transfected with FLAG-tagged Y1699C LRRK2 and myc-tagged constructs as indicated, and stained with antibodies against FLAG, myc and DAPI. Co-transfection efficiency was around 95 % in all cases. Cell boundaries (yellow) of co-transfected cells were determined by FLAG staining due to FLAG-Y1699C-LRRK2 expression. Scale bar, 10 µm. (**C**) Example of A549 cells co-transfected with FLAG-tagged Y1699C LRRK2 and the indicated myc-tagged constructs, and stained for FLAG, pericentrin and DAPI. Arrows point to centrosomes in transfected cells. Cell boundaries (yellow) were determined by FLAG staining due to FLAG-Y1699C-LRRK2 expression. Scale bar, 10 µm. (**D**) Quantification of the split centrosome phenotype in cells co-transfected with FLAG-tagged Y1699C LRRK2 and the indicated myc-tagged constructs. Around 30 transfected cells with duplicated centrosomes were analyzed for each condition. Bars represent mean ± s.e.m. (n=2 independent experiments; ctrl versus Y1699C-LRRK2, $p = 0.005$; Y1699C-LRRK2 versus Y1699C-LRRK2 + rootletin, $p = 0.032$; Y1699C-LRRK2 versus Y1699C-LRRK2 + Nek2a-dn, $p = 0.047$; Y1699C-LRRK2 versus Y1699C-LRRK2 + PP1α, $p = 0.026$; Y1699C-LRRK2 versus Y1699C-LRRK2 + PP1α-CA, $p = 0.025$); $^{**}p < 0.01$; $^{*}p < 0.05$.

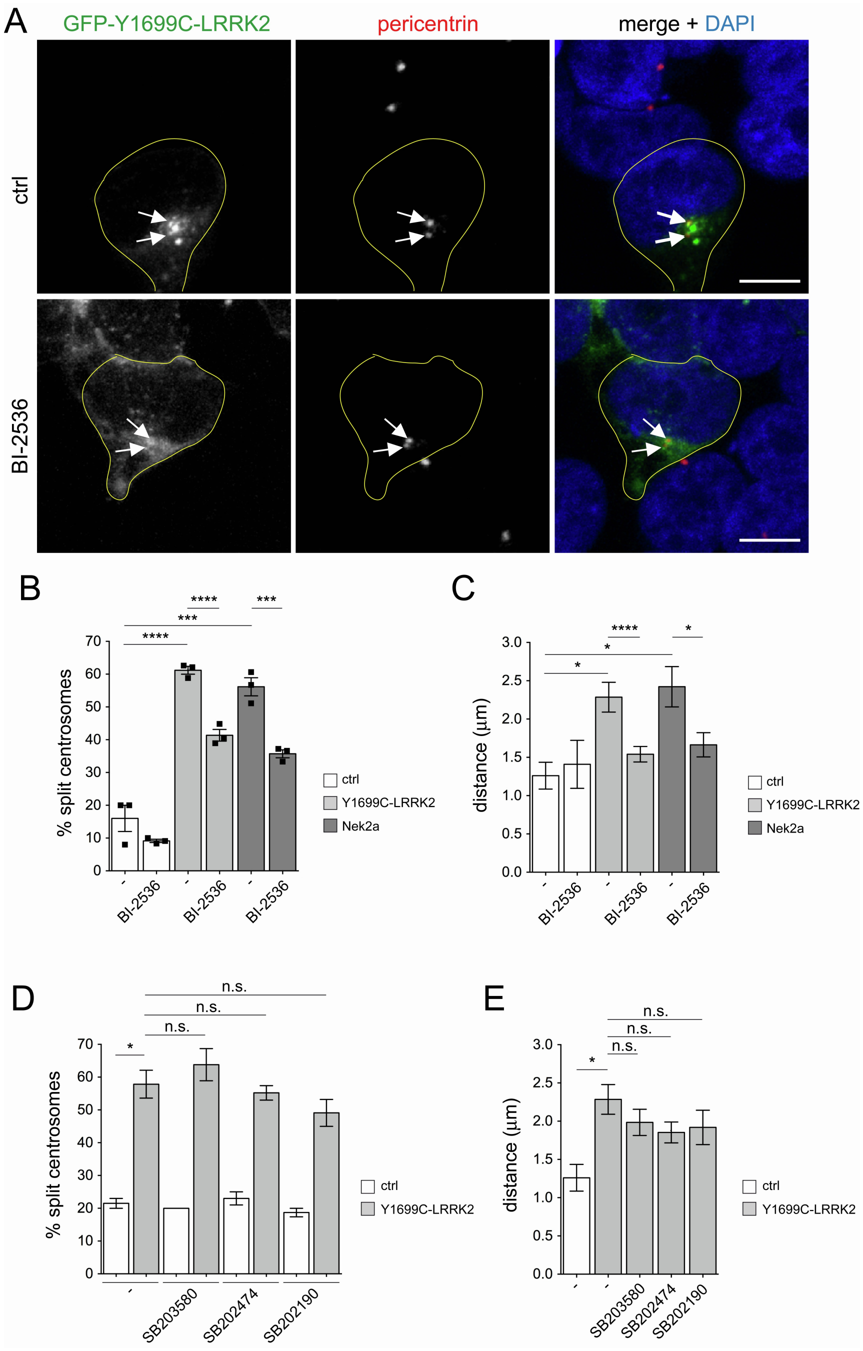

Figure S8. Plk1 inhibition partially reverts the pathogenic LRRK2-mediated centrosomal cohesion deficits. Related to Figure 3. (**A**) Example of HEK293T cells transfected with GFP-tagged Y1699C-LRRK2 and either left untreated (ctrl), or treated with the Plk1 inhibitor BI-2536 (100 nM, 2 h) before staining with pericentrin and DAPI. Arrows point to centrosomes in transfected cells. Cell boundaries (yellow) are shown and were determined due to GFP-tagged Y1699C-LRRK2 expression. Scale bar, 10 µm. (**B**) Quantification of the split centrosome phenotype in either non-transfected cells (ctrl), or cells transfected with GFP-tagged Y1699C-LRRK2 or with myc-tagged Nek2a as indicated, and either left untreated (-) or treated with BI-2536 as indicated. Bars represent

mean ± s.e.m. (n=3 independent experiments; ctrl versus Y1699C-LRRK2, $p < 0.001$; ctrl versus Nek2a, $p = 0.001$; Y1699C-LRRK2 versus Y1699C-LRRK2 + BI-2536, $p < 0.001$; Nek2a versus Nek2a + BI-2536, $p = 0.002$); ****$p < 0.001$; ***$p < 0.005$. (**C**) Same as in (B), but distances between duplicated centrosomes were measured from 30-70 cells per condition and experiment. Bars represent mean ± s.e.m. (ctrl versus Y1699C-LRRK2, $p = 0.016$; ctrl versus Nek2a, $p = 0.041$; Y1699C-LRRK2 versus Y1699C-LRRK2 + BI-2536, $p < 0.001$; Nek2a versus Nek2a + BI-2536, $p = 0.023$); ****$p < 0.001$; *$p < 0.05$. (**D**) HEK293T cells were transfected with GFP-tagged Y1699C-LRRK2 as indicated, and treated with 4 μM of SB203580 (p38β MAP kinase inhibitor), SB202474 (inactive structural analog), or SB202190 (p38α MAP kinase inhibitor) before analysis of centrosomal cohesion phenotype. Bars represent mean ± s.e.m. (n=2 independent experiments; ctrl versus Y1699C-LRRK2, $p = 0.015$); *$p < 0.05$. N.s., not significant. (**E**) Same as in (D), but distances between duplicated centrosomes were measured from 30-60 cells per condition and experiment. Bars represent mean ± s.e.m. (n=2 independent experiments; ctrl versus Y1699C-LRRK2, $p = 0.016$); *$p < 0.05$. N.s., not significant.

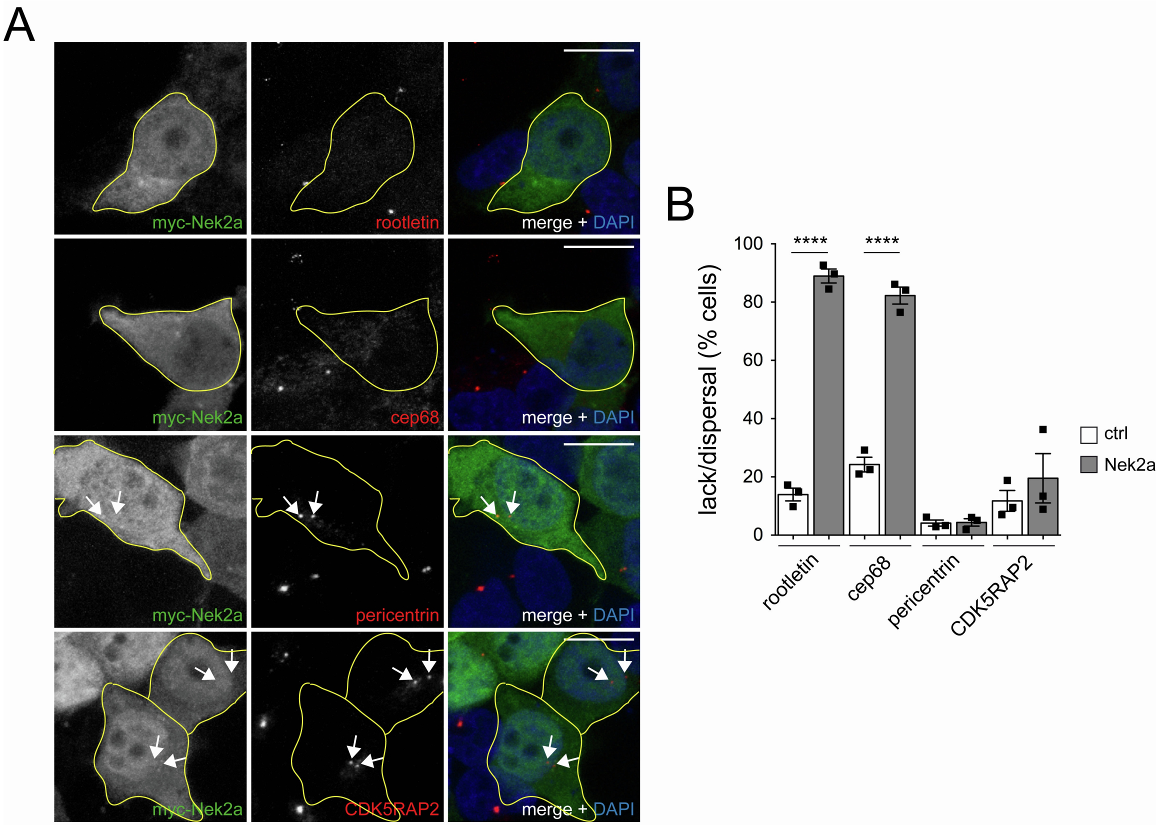

Figure S9. Nek2a expression causes loss/dispersal of rootletin and cep68 but not of CDK5RAP2 staining. Related to Figure 4. (**A**) Example of HEK293T cells transfected with myc-tagged Nek2a and stained for proteinaceous centrosomal linker proteins rootletin and cep68, centrosomal marker pericentrin, or CDK5RAP2 along with DAPI. Arrows point to centrosomes in transfected cells. Cell boundaries (yellow) are shown and were determined by myc staining due to myc-tagged Nek2a expression. Scale bar, 10 μm. (**B**) Quantification of the percentage of control or Nek2a-transfected cells displaying a lack of staining with antibodies against various proteins as indicated. Around 100-150 transfected cells were scored per condition and experiment. Bars represent mean ± s.e.m. (n=3 independent experiments; $p < 0.001$ in both cases); ****$p < 0.001$.

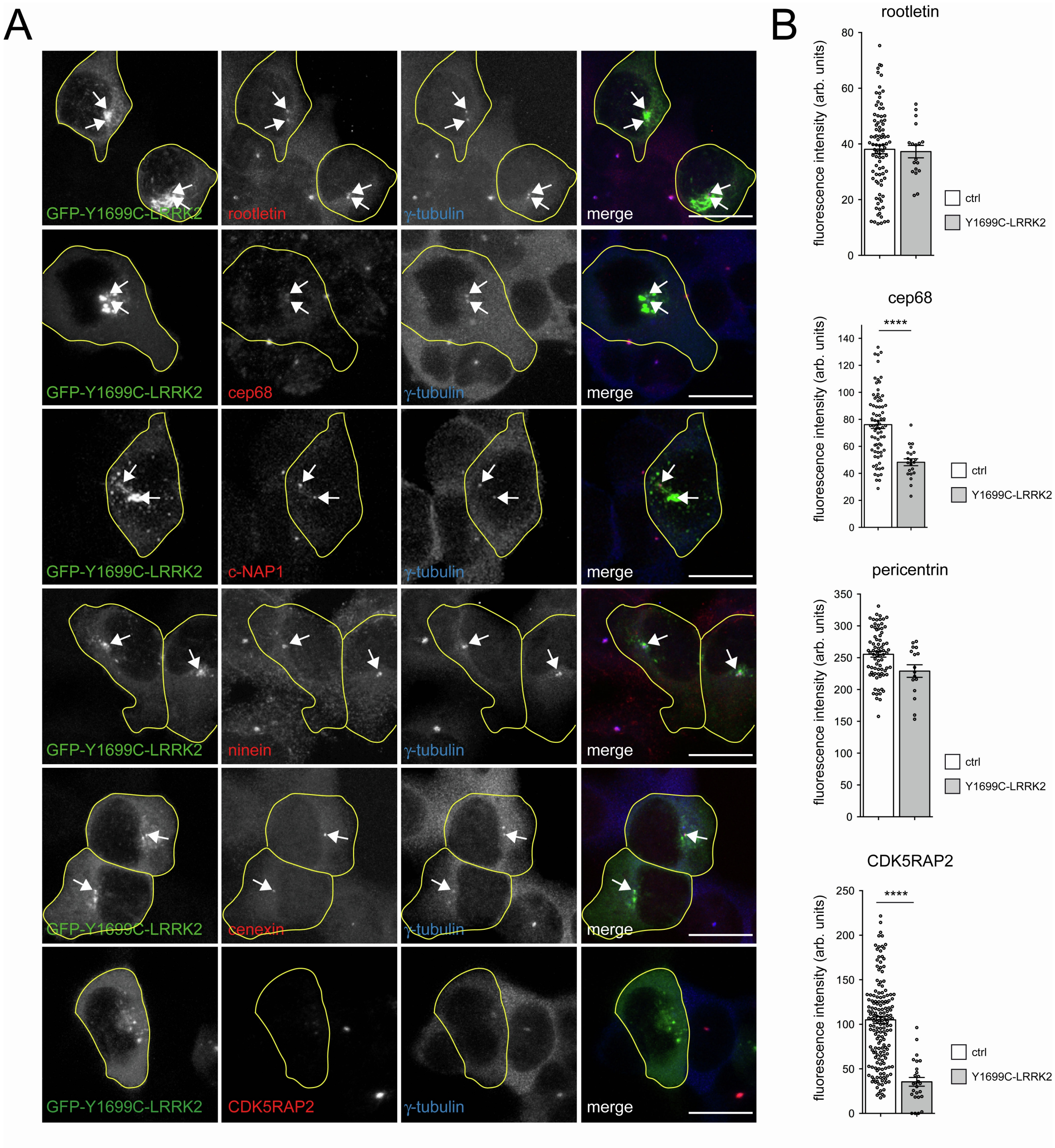

Figure S10. Pathogenic LRRK2 expression causes loss/dispersal of centrosomal CDK5RAP2. Related to Figure 4. (**A**) Example of HEK293T cells transfected with GFP-tagged Y1699C-LRRK2 and stained for various proteins as indicated, along with γ-tubulin as centrosomal marker. Arrows point to centrosomes as assessed by staining with the centrosomal marker γ-tubulin in transfected cells. Cell boundaries (yellow) are shown and were determined due to GFP-tagged Y1699C-LRRK2 expression. Scale bar, 10 μm. (**B**) A circle with diameter of 1.6 μm was placed on top of each centrosome as identified by γ-tubulin staining, and integrated fluorescence density of centrosomal rootletin, cep68, pericentrin and CDK5RAP2 quantified from non-transfected and transfected cells. Bars represent mean ± s.e.m. (n=16-177 cells per condition; $p < 0.001$ in both cases); ****$p < 0.001$.

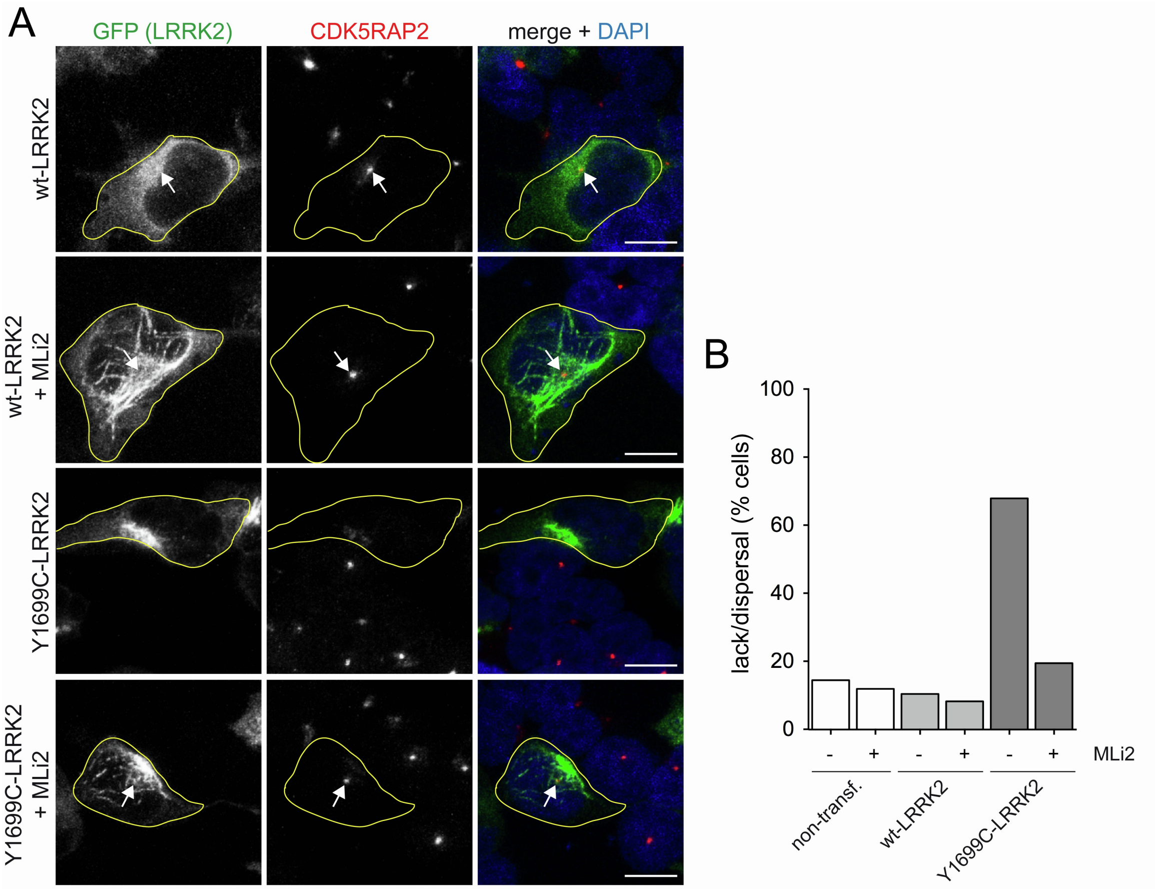

Figure S11. Pathogenic LRRK2 expression causes loss/dispersal of centrosomal CDK5RAP2. Related to Figure 4. (A) Example of HEK293T cells transfected with GFP-tagged wildtype or Y1699C-LRRK2, either in the presence or absence of 100 nM MLi2 for 2 h before processing for immunocytochemistry with a mouse monoclonal anti-CDK5RAP2 antibody and DAPI. Arrows point to CDK5RAP2 staining in transfected cells. Cell boundaries (yellow) are shown and were determined due to GFP-tagged Y1699C-LRRK2 expression. Scale bar, 10 μm. (**B**) Quantification of the percentage of control non-transfected, wildtype or Y1699C-LRRK2 transfected cells displaying a dispersal or a complete lack of staining with a mouse monoclonal anti-CDK5RAP2 antibody, either in the presence or absence of 100 nM MLi2 for 2 h before processing. Around 100-150 cells were scored per condition.

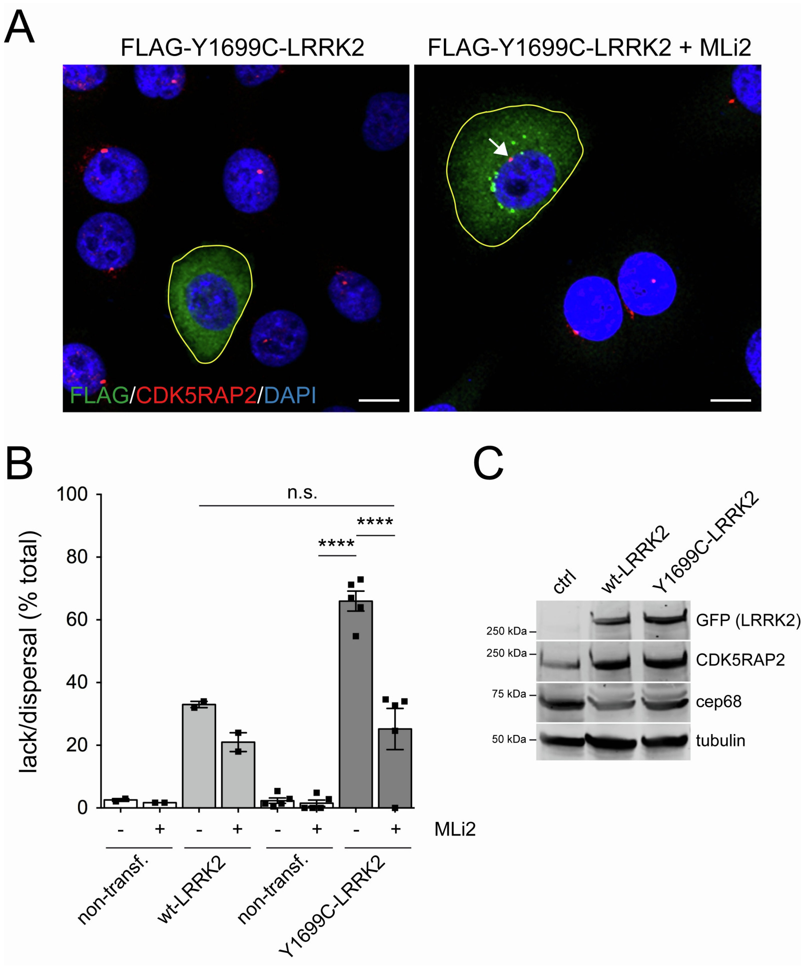

Figure S12. Pathogenic LRRK2 expression causes loss/dispersal of CDK5RAP2 staining in A549 cells. Related to Figure 4. (**A**) Example of A549 cells transfected with FLAG-tagged Y1699C-mutant LRRK2, treated with or without MLi2 (200 nM, 2 h) and stained for CDK5RAP2 and DAPI. Arrow points to CDK5RAP2 staining in transfected cell. Cell boundaries (yellow) are shown and were determined by FLAG staining due to FLAG-tagged Y1699C-LRRK2 expression. Scale bar, 10 μm. (**B**) Quantification of the percentage of control or Y1699C-LRRK2 transfected cells displaying a complete lack of staining with anti-CDK5RAP2 antibody. Around 40-50 transfected cells were scored per condition and experiment. Bars represent mean ± s.e.m. (n=2-5 independent experiments; $p < 0.001$ in both cases); ****$p < 0.001$. (**C**) HEK293T cells were either left untreated (ctrl), or transfected with GFP-tagged wildtype or Y1699C-LRRK2, and extracts blotted for GFP, CDK5RAP2, cep68 and tubulin as loading control.

Table S1. Detailed information on iPSC clones employed. Related to STAR Methods.

ID of Fibroblast	ID of iPSC clone	Diagnosis	Genotype LRRK2	Gender	Age of Biopsy (years)	Reprogramming method	PMID
SFC832	SFC832-03-06	PD	G2019S/WT	F	77	Cytotune1	32359446
SFC832	SFC832-03-06 LRRK2 WT/WT-C47	CRISPR/Cas-9 edited	WT/WT				32359446
SFC840	SFC840-03-03	Healthy	WT/WT	F	67	Cytotune1	26905200
SFC840	SFC840-03-03 LRRK2 WT/R1441C-H3	CRISPR/Cas-9 edited	R1441C/WT				This study
